# Impact of in vitro phytohormone treatments on the metabolome of the leafy liverwort *Radula complanata* (L.) Dumort

**DOI:** 10.1007/s11306-023-01979-y

**Published:** 2023-03-09

**Authors:** Kaitlyn Blatt-Janmaat, Steffen Neumann, Florian Schmidt, Jörg Ziegler, Yang Qu, Kristian Peters

**Affiliations:** 1 Department of Chemistry, University of New Brunswick, Fredericton, E3B 5A3 NB Canada; 2 Bioinformatics and Scientific Data, Leibniz Institute of Plant Biochemistry, Weinberg 3, 06120 Halle (Saale), Germany; 3 German Centre for Integrative Biodiversity Research (iDiv) Halle-Jena-Leipzig, Puschstraße 4, 04103 Leipzig, Germany; 4 Molecular Signal Processing, Leibniz Institute of Plant Biochemistry, Weinberg 3, 06120 Halle (Saale), Germany; 5 Institute of Biology/Geobotany and Botanical Garden, Martin Luther University Halle-Wittenberg, Am Kirchtor 1, 06108 Halle (Saale), Germany

**Keywords:** Untargeted metabolomics, Liverworts, Phytohormones, LC-MS, Metabolite identification, Compound classification

## Abstract

**Introduction:**

Liverworts are a group of non-vascular plants that possess unique metabolism not found in other plants. Many liverwort metabolites have interesting structural and biochemical characteristics, however the fluctuations of these metabolites in response to stressors is largely unknown.

**Objectives:**

To investigate the metabolic stress-response of the leafy liverwort *Radula complanata*.

**Methods:**

Five phytohormones were applied exogenously to in vitro cultured *R. complanata* and an untargeted metabolomic analysis was conducted. Compound classification and identification was performed with CANOPUS and SIRIUS while statistical analyses including PCA, ANOVA, and variable selection using BORUTA were conducted to identify metabolic shifts.

**Results:**

It was found that *R. complanata* was predominantly composed of carboxylic acids and derivatives, followed by benzene and substituted derivatives, fatty acyls, organooxygen compounds, prenol lipids, and flavonoids. The PCA revealed that samples grouped based on the type of hormone applied, and the variable selection using BORUTA (Random Forest) revealed 71 identified and/or classified features that fluctuated with phytohormone application. The stress-response treatments largely reduced the production of the selected primary metabolites while the growth treatments resulted in increased production of these compounds. 4-(3-Methyl-2-butenyl)-5-phenethylbenzene-1,3-diol was identified as a biomarker for the growth treatments while GDP-hexose was identified as a biomarker for the stress-response treatments.

**Conclusion:**

Exogenous phytohormone application caused clear metabolic shifts in *Radula complanata* that deviate from the responses of vascular plants. Further identification of the selected metabolite features can reveal metabolic biomarkers unique to liverworts and provide more insight into liverwort stress responses.

**Supplementary Information:**

The online version contains supplementary material available at 10.1007/s11306-023-01979-y.

## Introduction

Liverworts (Marchantiophtya) are a group of non-vascular plants found in almost every terrestrial ecosystem around the world (Söderström et al., 2016). Evolutionarily, bryophytes as a whole comprise a monophylic group that is placed between algae and pteridophytes (Asakawa et al., 2013; Harris et al., 2020). Despite their relatively simple physical morphology, liverworts are incredibly chemically diverse, largely due to the presence of oil bodies (He et al., 2013). These organelles are responsible for the sequestration, storage, and synthesis of the majority of the specialized metabolites produced by liverworts (Flegel & Becker, 2000; Suire et al., 2000; Tanaka et al., 2016). Oil bodies are responsible for the high chemodiversity of liverworts, which has resulted in them being more chemically studied than any other group of bryophyte (Asakawa & Ludwiczuk, 2018). Metabolites of bryophytic origin have recently garnered increased attention due to their potential cytotoxicity against human cancer cell lines (Dey & Mukherjee, 2015), herbicidal activity (Zhang et al., 2019a, b) and fungicidal activity (Commisso et al., 2021).

In this study, we investigate shifts in the metabolites in the leafy liverwort *Radula complanata* (L.) Dumort. due to exogenous phytohormone application to plants grown in vitro at the global metabolic level. The following hormones were chosen as they are known to induce large metabolic changes in vascular plants. Their function in bryophytes is yet largely unknown. Two growth inducing hormones (NAA and BAP) were chosen to stimulate vegetative plant growth while three stress-response phytohormones (MeJA, SA, and AA) were applied to induce stress response metabolites. NAA, 1-napthaleneacetic acid, is an auxin involved in tuning plant growth and development (Singh et al., 2021) while BAP, 6-benzylaminopurine, is a cytokinin; an important regulatory hormone involved in plant growth (Wu et al., 2021). Methyl jasmonate (MeJA), abscisic acid (AA), and salicylic acid (SA) are key signaling hormones that modulate plant responses to biotic or abiotic stressors. While liverworts respond to exogenous AA application, they do not produce AA endogenously. Instead, they utilize lunularic acid which serves as a bibenzyl growth inhibitor (Asakawa et al., 2010; Pryce, 1971a, b). Previous work by Kageyama et al. has demonstrated that (bis)bibenzyl production was increased through AA application, suggesting that stress-response hormones can be applied to stimulate specialized metabolite production in liverworts (Kageyama et al., 2015). Jasmonic acid is another phytohormone not produced endogenously in liverworts, with the precursor dn-OPDA instead activating the COI1 receptor in the jasmonate signaling pathway (Monte et al., 2019).

To examine the full range of specialized metabolites in liverworts, untargeted metabolomics can be utilized to monitor the changes of thousands of metabolites without the necessity of isolating them (Peters et al., 2021). This approach could also provide insight into the biosynthetic pathways by monitoring potential intermediates of compounds of interest (Fiehn, 2002; Jones et al., 2013). Recent work has demonstrated that untargeted metabolomics is an extremely effective tool for the analysis of the chemodiversity in bryophytes, however there are very few studies to date that utilize this methodology (Lu et al., 2021; Peters et al., 2019; Peters, Worrich, Peters et al. 2018a, b). In addition, we performed chemodiversity analyses to characterize the large number of metabolite features impacted by the different hormone treatments at a global level. Chemodiversity analysis can reveal the richness, relative abundance, and the structural dissimilarity of metabolite features produced by *R. complanata* in the different hormone treatments (Peters et al., 2022; Petrén et al., 2022). To complement the untargeted approach, targeted metabolomics can also be applied *post-hoc* to quantitatively analyze specific metabolites, however a standard of the compound is usually necessary (Commisso et al., 2021). Due to the necessity of authentic standard samples, this analysis can be difficult for metabolites that are not commercially available.

In this study, the impact of various hormone treatments on the metabolome of *Radula complanata* was investigated at the global metabolic level using ultra-performance liquid chromatography coupled to electrospray ionization quadrupole time-of-flight mass spectrometry (UPLC/ESI-QTOF-MS) with data-dependent acquisition (DDA-MS). The overall chemodiversity of in vitro cultivated *Radula complanata* was examined and the hormone response was tracked through the selection of features that varied across different phytohormone treatments. The selected features were tentatively classified to observe how the different chemical classes fluctuated in response to exogenous phytohormone application.

## Methods

The sample material for this work was utilized in a separate study conducted by our group that focused on identifying variations in bibenzyl metabolites specifically (Blatt-Janmaat et al., 2022). The methodology pertaining to plant material preparation, metabolite extraction, and LC analysis (Sect. 2.1 through 2.6) are reported in that study and briefly summarized here.

### Plant preparation and hormone treatments

Sterile *Radula complanata* protonema were germinated from spores on 20-20-20 agar plates and cultivated to obtain mature gametophytes. Voucher specimens of environmental samples used for spore collection have been deposited at the Connell Memorial Herbarium at the University of New Brunswick, Fredericton under the accession numbers 69,083 and 69,084.

### Hormone treatments

Mature gametophytes were placed on 20-20-20 agar plates supplemented with hormones and grown under full spectrum growth lights. Stress-response hormones included methyl jasmonate (MeJA, Sigma), abscisic acid (AA, Sigma), and salicylic acid (SA, Sigma) while growth hormones included 1-napthaleneaceticacid (NAA, Sigma), and 6-benzylaminopurine (BAP, Sigma). Control samples (no methanol, no hormones) were prepared for both the stress-response and growth groups. The stress-response hormones were grown for 4 months while the growth hormone treatments were grown for 3 months. Plants were weighed and stored at -80 °C until further processing. Harvesting yielded the following samples: AA1, AA10, AA100, MeJA1, MeJA10, MeJA100, SA1, SA10, SA100, Stress Control, BAP1, BAP10, BAP100, NAA1, NAA10, NAA100, Growth Control. Statistical analyses were conducted in R and significant changes were determined by a one-way ANOVA and a Tukey HSD post-hoc test. Treatment fresh weights were normalized with Eq. (1) for data visualization.

1 $$Normalized value= \frac{(Sample-Control)}{Control}$$

### Metabolite extractions

We followed extraction procedures for LC-MS originally established by Böttcher et al. (Böttcher et al., 2009) for vascular plants and modified slightly by Peters et al. for bryophytes (Peters, Gorzolka, et al., 2018). This method was observed to give robust results regarding our targeted compound classes (Lu et al., 2021). Frozen plants were homogenized and extracted with 1 mL of cold 80:20 MeOH:H_2_O supplemented with 5 µM Kinetin (Sigma), 5 µM Biochanin A (Sigma), and 5 µM N-(3-Indolylacetyl)-L-alanine (Sigma). For LC-MS analysis, samples were reconstituted to 10 mg fresh weight/100 µL with 80:20 MeOH:H_2_O.

### Chromatographic separation and untargeted mass-spectrometry

Samples were separated and analyzed with a Bruker Elite HPLC equipped with a Nucleodur X18 Gravity-SB column (1.8 μm 100 × 2 Macherey Nagel, Dueren, Germany) coupled to a Bruker TIMS-TOF (timsTOF Pro, Bruker, Bremen, Germany). 0.1% aqueous formic acid and acetonitrile were utilized as the mobile phase with a flow rate of 0.5 mL/min. 2 µL was injected per sample and the injection chamber was maintained at 4 °C. Separate injections were performed for analysis in positive and negative mode and ionized with electrospray ionization (ESI). Data was collected in data-dependent acquisition (DDA-MS) mode (also called auto-MS/MS by the vendor) with the instrument settings reported in Blatt-Janmaat et al. (Blatt-Janmaat et al., 2022). Calibration for both modes was completed with Na-Formate.

To ensure consistency throughout the campaign, a quality control (QC) sample was injected every 8 samples to check retention time drift and to detect potential carryover in the column. The QC sample was composed of the three internal standard compounds (Biochanin A, Kinetin, and N-(3-Indolylacetyl)-L-alanine) as well as six bibenzyl metabolites (Radulanin A, Radulanin H, Radulanin L, 4-prenyldihydropinosylvin, 3,5-dihydroxy-6-carbomethoxy-2-(3-methyl-2-butenyl)bibenzyl, and 2-(3,7-Dimethylocta-2,6-dienyl)-5-(2-phenylethyl)benzene-1,3-diol) that were previously isolated from *Radula complanata*.

### Raw data acquisition

Raw LC-MS files (Bruker Daltonics .d format) were converted to .mzML with MSConvertGUI version 3 from the ProteoWizard software suite (available here: https://proteowizard.sourceforge.io/download.html) (Chambers et al., 2012). Raw data has been uploaded to MetaboLights as MTBLS3563 (www.ebi.ac.uk/metabolights/MTBLS3563) (Haug et al., 2020).

### Chromatographic peak detection in untargeted LC-MS

Chromatographic peak detection was performed in R 4.1.1 (available from https://cran.r-project.org/) with the XCMS 3.14.1 package (Smith et al., 2006). Parameter optimization with IPO (Libiseller et al., 2015) was conducted and additional manual adjustments were made based on instrument knowledge. Data was restricted to 0 to 1020 s and the *centWave* algorithm was applied for peak detection (Tautenhahn et al., 2008). Peaks were grouped and retention time corrected using the *adjustRtime* function in XCMS. After retention time correction, samples were regrouped and peaks were filled with the *fillChromPeaks* function in XCMS. All parameters can be found in Blatt-Janmaat et al. (Blatt-Janmaat et al., 2022).

### Data treatment

To assess the quality of the obtained spectral data, EICs for the three internal standards and six previously identified bibenzyls were extracted and manually analyzed for peak shape and retention time shifts. After an initial quality check of the data, positive and negative data sets were processed independently and merged into one feature table once features were detected and annotated. Several tables were constructed from the original feature table based on various constraints. A presence/absence table was constructed to determine if a peak was present in the MS1 data and a cut-off at 0.01% of the max peak intensity was applied. A table containing features that were only present in each treatment and a compound table containing only features for which there were MS2 spectra available was constructed. Data tables are available in MetaboLights as MTBLS3563. In preparation for statistical analysis, missing MS1 data was replaced with the median intensity value and the matrix was log transformed. After transformation, useable MS2 spectra were extracted from MS1 spectra, imported to SIRIUS 4.9.6 and the molecular formula was determined.

### Statistical analyses

All statistical tests were conducted in R. To assess the variance of different explanatory factors such as experimental design, a variation partitioning was conducted using the *varpart* function from the vegan package. Principal Component Analysis (PCA) using the *prcomp* function was performed to visualize sample separation. To test which PC axes explain more variance than would be expected by randomly dividing the variance into parts, the broken stick test was performed using the function *screeplot* and *bstick* from the R package *vegan*. The following chemodiversity measures were calculated: the overall feature richness by summing the counts of detected features per profile, the Shannon diversity index (H’), the Pielou’s evenness index (J) (Peters et al., 2022), and the functional Hill diversity which also takes the dissimilarity of features into account (Petrén et al., 2022). Boxplots were drawn using the *boxplot* function in R and a one-way ANOVA followed by a Tukey Honestly Significant Difference (HSD) post-hoc test using the *multcomp* package to identify significant differences. Chemodiversity boxplots were produced using the built-in function *boxplot* in R. To determine whether any factor varied across the different treatments, a one-way ANOVA followed by a Tukey HSD post-hoc test was conducted using the functions *glht* and *cld* from the *multcomp* R package.

Selection of variables that contribute significantly to the examined effects was accomplished with applying the BORUTA algorithm on Random Forest prediction models. BORUTA is an algorithm that eliminates variables that do not contribute to the examined factor by performing permutation tests on the variable importance of different Random Forest models (Kursa & Rudnicki, 2010). The following arguments were used for the function BORUTA: x = feature_matrix, y = compound_matrix, mcAdj = TRUE, maxRuns = 10,000, doTrace = 0, holdHistory = TRUE, getImp = getImpRfZ. BORUTA already provides out-of-bag (OOB) errors internally and validates selected variables according to paired t-tests (Kursa & Rudnicki, 2010). To provide comparable classification metrics, a regression tree was built post-hoc using the function *rtree*. Common performance metrics used in bioinformatics such as R-squared (R^2^) and Root Mean Square Error (RMSE) were calculated by creating a dummy object with similar data structure than the R package caret. Then, metrics were calculated on the dummy object using the function *postResample* and *multiClassSummary* from the caret package. For the final BORUTA, the compound table was normalized with respect to the control with Eq. (1).

To visualize the selected variables, heatmaps were implemented using the function *heatmap.2* in R. Columns and rows were clustered using a Euclidean distance measure. For the samples, a complete method was used to agglomerate the distances and the Ward.D method was used for the features. To test the selected variables for relation to pathways, a molecular network was calculated and visualized using the functions *molNet* and *molNetPlot* from the chemodiv package.

### Compound classification

Peaks were classified and tentatively annotated with SIRIUS (Dührkop et al., 2019), ZODIAC (Ludwig et al., 2020), CSI:FingerID (Dührkop et al., 2015; Hoffmann et al., 2021), and CANOPUS (Djoumbou Feunang et al., 2016; Dührkop et al., 2021) using ClassyFire and NPClassifier (Djoumbou Feunang et al., 2016; Kim et al., 2021) based on MS2 fragmentation patterns using SIRIUS software version 5.6.2. Default settings were used for SIRIUS, ZODIAC, and CSI:FingerID, however only formulas from natural-product based databases were considered (Bio Datadase, Biocyc, CHEBI, COCONUT, EcoCyc Mine, CNPS, HMDB, KEGG, KEGG Mine, KNApSAcK, Natural Products and Plantcyc) for CANOPUS in the SIRIUS software suite. To tentatively identify a compound, a combination of COSMIC and ZODIAC scores were considered. Manual analysis of the matching substructures was conducted before structural assignment was made. If the fragmentation pattern did not match the structures proposed by CSI:FingerID, matching fragments from the proposed structures were considered when the class was assigned using CANOPUS. If the ZODIAC score was < 50%, no tentative identification was made. If the SIRIUS score was < 50% with no accompanying ZODIAC score, no identification was made. For an overview of the detected compound classes, a sunburst plot of all classified features was constructed.

### Results

### Vegetative growth

**Fig. 1:**
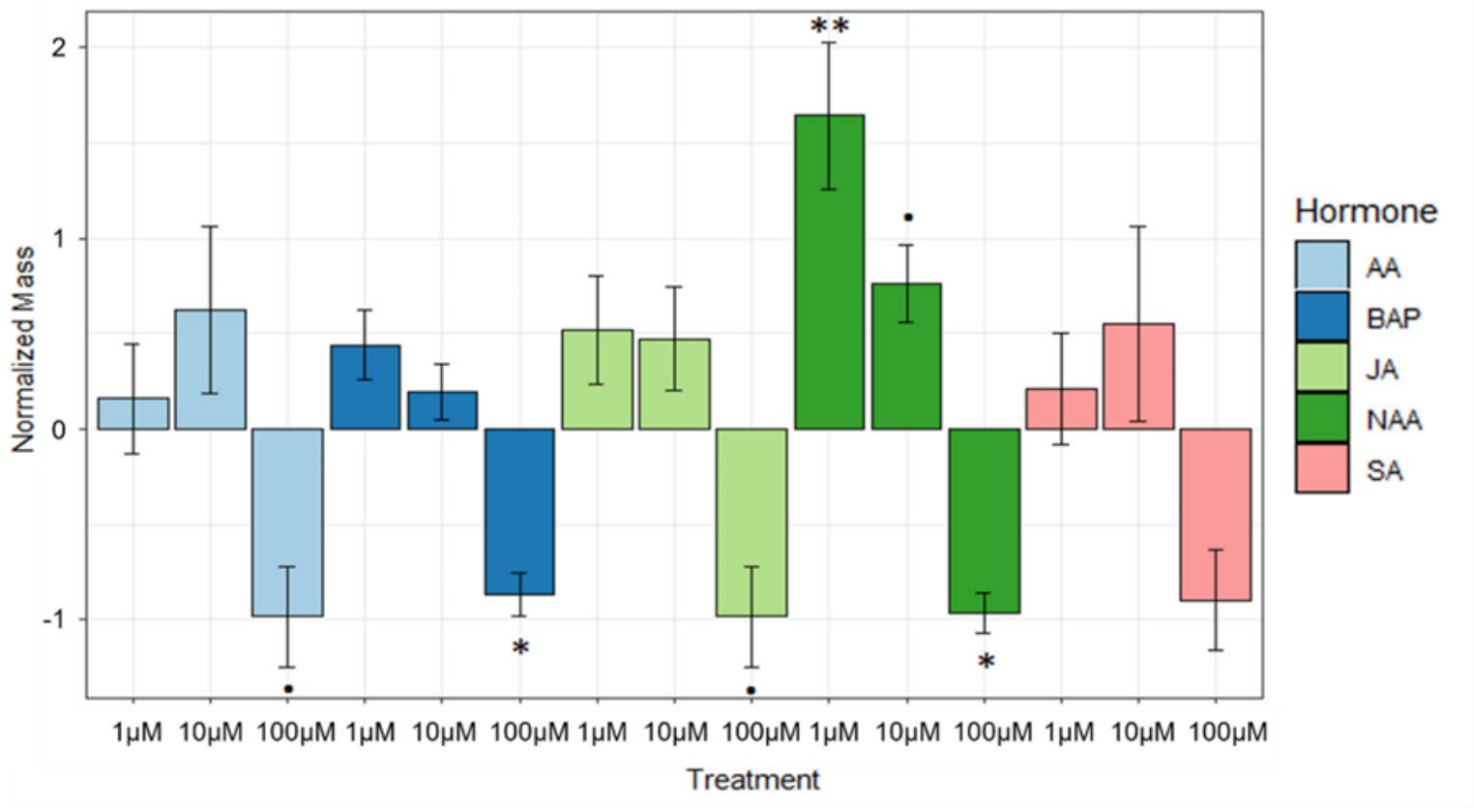
Normalized fresh weights of hormone treatments. Significance values were determined with comparison to the respective control. P-values between concentrations were determined by t-test while significance with respect to the control was determined by a one-way ANOVA/Tukey HSD post-hoc test. Significance codes: 0 ‘***’ 0.001 ‘**’ 0.01 ‘*’ 0.05 ‘.’ 0.1

First, we determined vegetative growth with regard to the different hormone treatments (Fig. 1). It was observed that the 100 µM treatments of all hormones significantly reduced the growth of the plants with the exception of SA (AA: *p* = 0.071479, BAP: *p* = 0.040255, MeJA: *p* = 0.07149, NAA: *p* = 0.019643, SA: *p* = 0.1269). A significant increase in vegetative growth was observed with NAA at 1 µM (*p* = 0.000159) and only a slight increase at 10 µM (*p* = 0.085448) concentrations but no significant increase was observed for any other treatments. Due to the significant reduction of growth in the 100 µM treatments, there was not enough plant material for further analysis.

### Feature grouping and chemodiversity

**Fig. 2:**
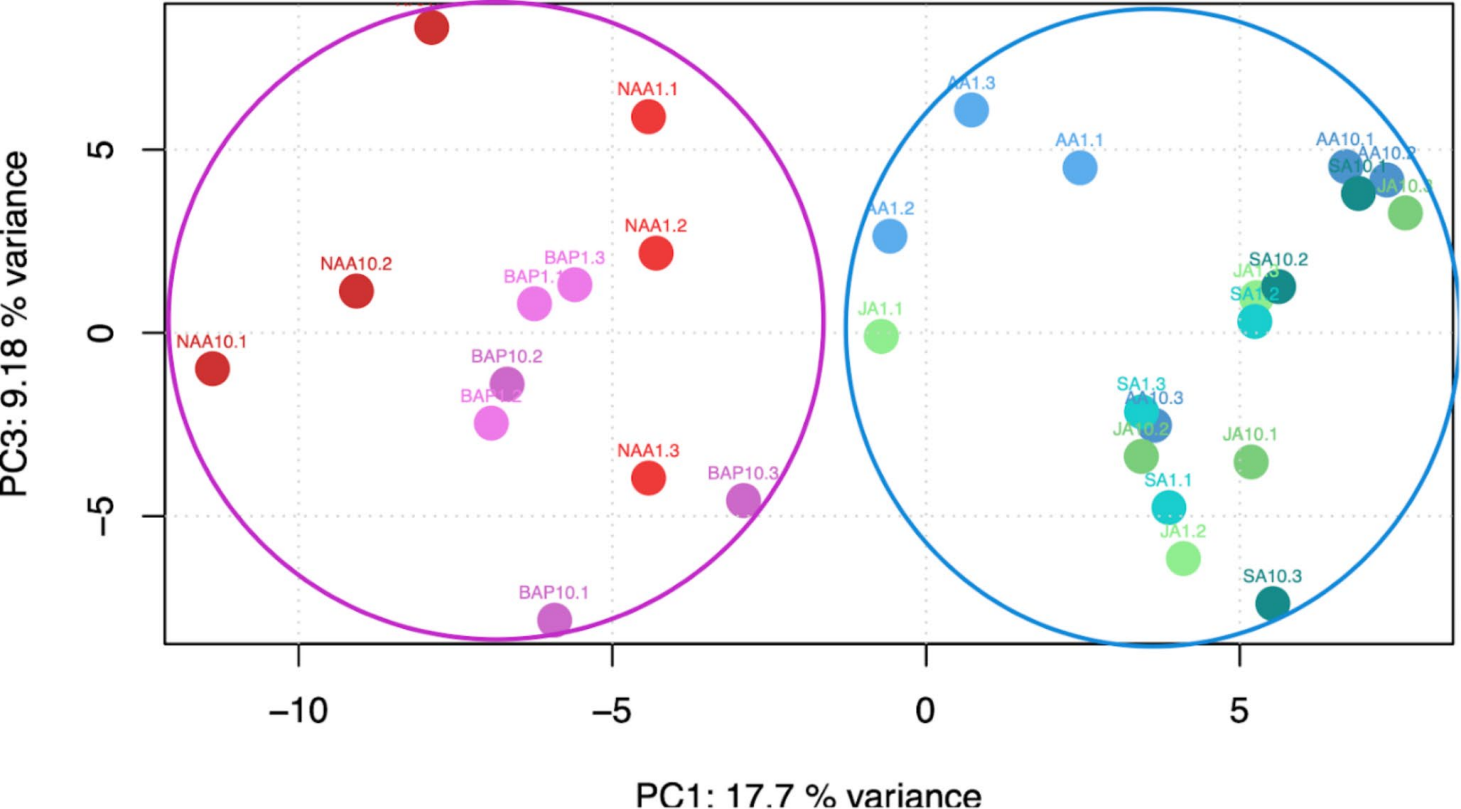
PCA of the normalized feature table. Circles were added manually with growth treatments (coloured pink-purple) circled in pink and stress-response treatments (coloured blue-green) circled in blue. As the broken stick test revealed that the PC1 and PC3 axes explained more variance than would be expected by randomly dividing the variance into parts, PC1 and PC3 were chosen for the plot. Results of the broken stick test are available in the Supplement and Zenodo

To assess general statistical importance of hormone treatments in the metabolite profiles, a Principal Component Analysis (PCA) was performed on the normalized data (Fig. 2). The hormone type appeared to be responsible for the variation observed in PC1 (which explained 17.7%) while variation between the specific phytohormones and the treatment concentrations appeared to be responsible for PC2 (9.18% explained variance). Overall, a clear separation between growth hormones (pink-purple) and stress-response hormones (blue-green) was observed. A PCA of the full feature table was also conducted and a similar separation was observed (Supplementary Information).

**Fig. 3:**
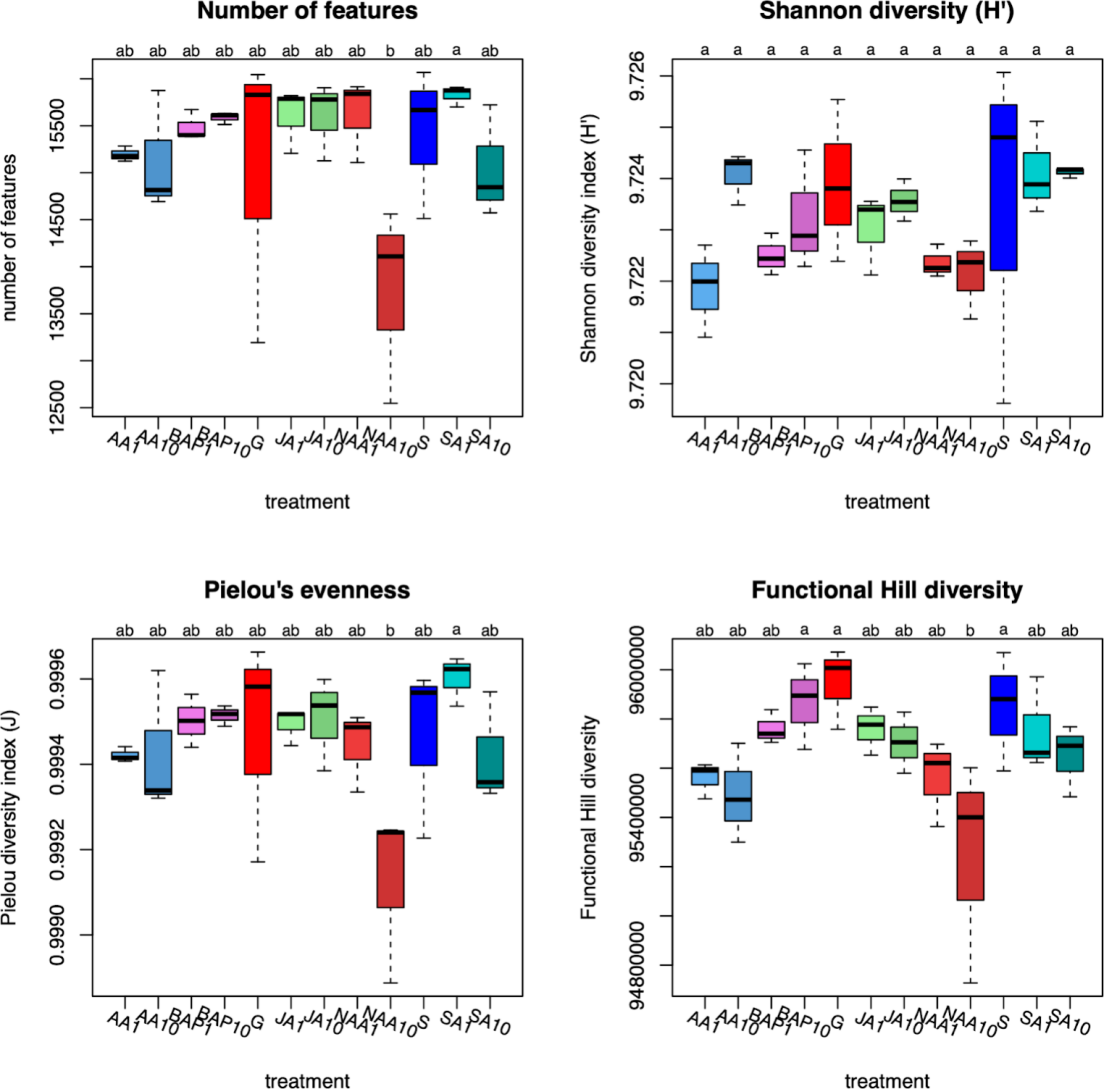
Diversity indices of the features detected in the MS1 data. A: number of features, B: Pielou’s evenness (J), C: Shannon diversity Index (H’). D: Functional Hill diversity. S: stress-response control, G: growth control

Analysis of the chemodiversity showed unique patterns with respect to the features detected in each treatment (Fig. 3). The number of features detected was consistent between all the treatments except for NAA10 which had significantly fewer features (Fig. 3a). The Pielou’s evenness (J) for all treatments was consistent, with the exception of SA1 and NAA10 which had significantly higher and lower J values, respectively (Fig. 3b). No significant differences were observed in the Shannon diversity (H’) (Fig. 3c). Determining the Functional Hill diversity, which also does take the dissimilarity of features into account (Petrén et al., 2022), showed that the significantly lower diversity in NAA10 was explained by structurally similar features with less abundance than the other treatments and a slightly higher dissimilarity of features in BAP10, G, and S (Fig. 3d).

### Compound classification

**Fig. 4:**
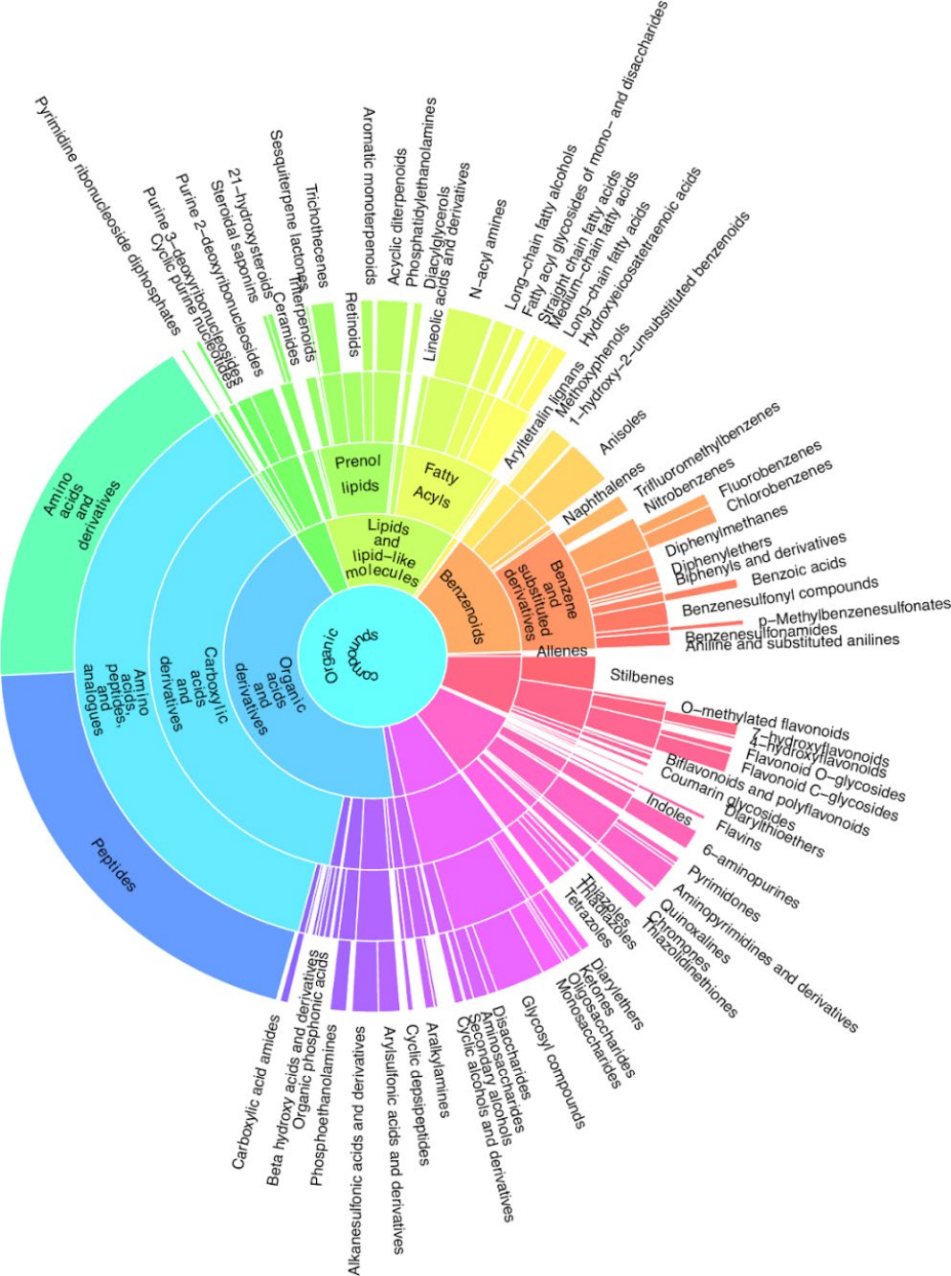
Sunburst plot showing an overview on the richness of classified metabolite compounds. Broad compound classes are represented in the center while specific classifications are represented on the exterior. Colours correspond to the assigned classes. Due to readability the names of some classes were removed from the plot. An interactive zoomable plot is available in the supplementary vignettes and on Zenodo

A total of 211 compound classes were identified in the positive and negative ion modes. To visualize the compound class diversity, a sunburst plot was conducted (Fig. 4). The most prominently detected classes overall were carboxylic acids and derivatives (mainly due to amino acids, peptides, and analogues), followed by benzene and substituted derivatives, fatty acyls (largely fatty amides), organooxygen compounds (mostly carbohydrates and carbohydrate conjugates), prenol lipids (mostly diterpenoids, retinoids, and sesquiterpenoids), and flavonoids (mostly flavonoid glycosides and hydroxyflavonoids). A large number of features were also classified as stilbenes, the chemical class represented in the ClassyFire chemical ontology that encompasses the characteristic bibenzyls found in *Radula* spp. Known compounds from liverworts were tentatively annotated and are listed in Table 1.

**Table 1:** Tentatively annotated liverwort specialized metabolites. Full details are found in the Supplementary Information

Compound	Formula	Molar Mass	Ionization	Tentative Feature
Bisabola-1,3,5,7(14),10- pentaene	C15H20	200.32	Positive	FT0671, FT0672
Ar-tenuifolene	C15H20	200.32	Positive	FT0671, FT0672
Eudesma-1,4(15)-11- triene	C15H22	202.23	Positive	FT0692
Myli-4(15)-ene	C15H22	202.33	Positive	FT0692
Cis-calamenene	C15H22	202.33	Positive	FT0692
Cuparene	C15H22	202.33	Positive	FT0692
Xanthorrizol	C15H22O	218.33	Positive	FT0828 - FT0832
2-cuparenol	C15H22O	218.33	Positive	FT0828 - FT0832
Cyclocolorenone	C15H22O	218.33	Positive	FT0828 - FT0832
β-herbertenol	C15H22O	218.33	Positive	FT0828 - FT0832
Trans-Nerolidol	C15H26O	222.37	Positive	FT0861
(E)-farnesol	C15H26O	222.37	Positive	FT0861
3-[2-(3-Methoxyphenyl)ethyl]phenol	C15H16O2	228.29	Positive	FT0923, FT0925
3,4′-Dimethoxybibenzyl	C16H18O2	242.31	Positive	FT1057, FT1059
1,2-Bis(3-methoxyphenyl)ethane	C16H18O2	242.32	Positive	FT1057, FT1059
Lunularic acid	C15H14O4	258.1	Negative	FT0814-FT0820
Radulanin A	C19H20O2	280.37	Positive	FT1451, FT1454, FT1458
2,2-Dimethyl-5-hydroxy- 7-(2-phenylethyl)- chromene*	C19H20O2	280.4	Positive	FT1454, FT1458
4-(3-Methyl-2-butenyl)-5-phenethylbenzene-1,3-diol	C19H22O2	282.38	Positive	FT1480, FT1483, FT1484, FT1487
			Negative	FT1001, FT1008, FT1009, FT1011
4-Prenyldihydropinosylvin	C19H22O2	282.38	Positive	FT1480, FT1483, FT1484, FT1487
			Negative	FT1001, FT1008, FT1009, FT1011
Radulanin A methyl ether	C20H22O2	294.39	Positive	FT1623, FT1624, FT1625, FT1626, FT1627
			Negative	FT1111, FT1112
8-[2-(4-Hydroxyphenyl)ethyl]-3-methyl-2,5-dihydro-1-benzoxepin-6-ol	C19H20O3	296.37	Negative	FT1132, FT1133, FT1135, FT1136, FT1139, FT1140, FT1141, FT1142, FT1143, FT1144, FT1147
5-Methoxy-2-(3-methylbut-2-en-1-yl)-3-(2-phenylethyl)phenol	C20H24O2	296.41	Positive	FT1658, FT1660
			Negative	FT1146, FT1148
4-(3-Methyl-2-Butenyl)-5-(2-Phenylethyl)-3-Methoxyphenol	C20H24O2	296.41	Positive	FT1658, FT1660
			Negative	FT1146, FT1148
2-[(3,3-Dimethyloxiran-2-yl)methyl]-5-(2-phenylethyl)benzene-1,3-diol	C20H24O2	296.41	Positive	FT1658, FT1660
			Negative	FT1146, FT1148
3-Methoxy-5-(2-phenylethyl)-2-prenylphenol	C20H24O2	296.41	Positive	FT1658, FT1660
			Negative	FT1146, FT1148
2-[(3,3-Dimethyloxiran-2-yl)methyl]-5-(2-phenylethyl)benzene-1,3-diol	C19H22O3	298.38	Negative	FT1167, FT1168
Kaempferol 3-methyl-ether	C16H12O6	300.26	Negative	FT1200, FT1201
2,2-Dimethyl-5-hydroxy-7-(2-phenylethyl)-2 H-1-benzopyran-6-carboxylic acid	C20H20O4	324.38	Negative	FT1483, FT1484, FT1485, FT1486, FT1489, FT1491, FT1494, FT1496
Radulanin E	C20H20O4	324.38	Negative	FT1483, FT1484, FT1485, FT1486, FT1489, FT1491, FT1494, FT1496
Radulanin H	C20H20O4	324.4	Positive	FT2017 - FT2020
			Negative	FT1484-1486, FT1489-1494, FT1496

### Feature selection and variation between hormone treatments

**Fig. 5:**
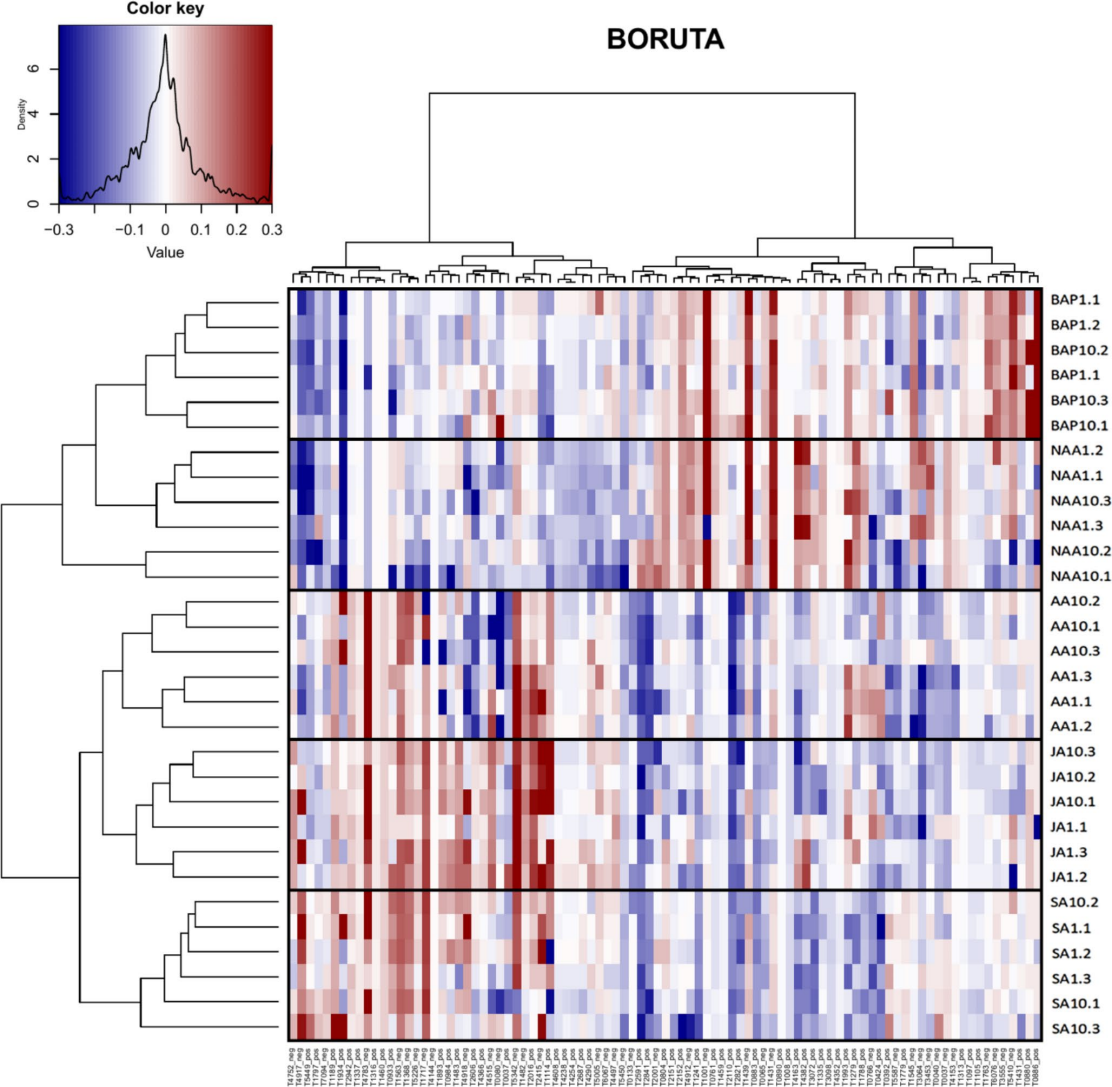
Heatmap showing the 91 partly annotated variables selected by the normalized BORUTA. The y-axis displays the clustering of samples and the x-axis displays the clustering of selected features. R2 = 0.75, RMSE = 1.095445, MAE = 0.8. Black boxes were drawn to better differentiate the shifts in the samples. Blue indicates that a feature was produced less than the control and red indicates that a feature was produced more than the control

**Fig. 6:**
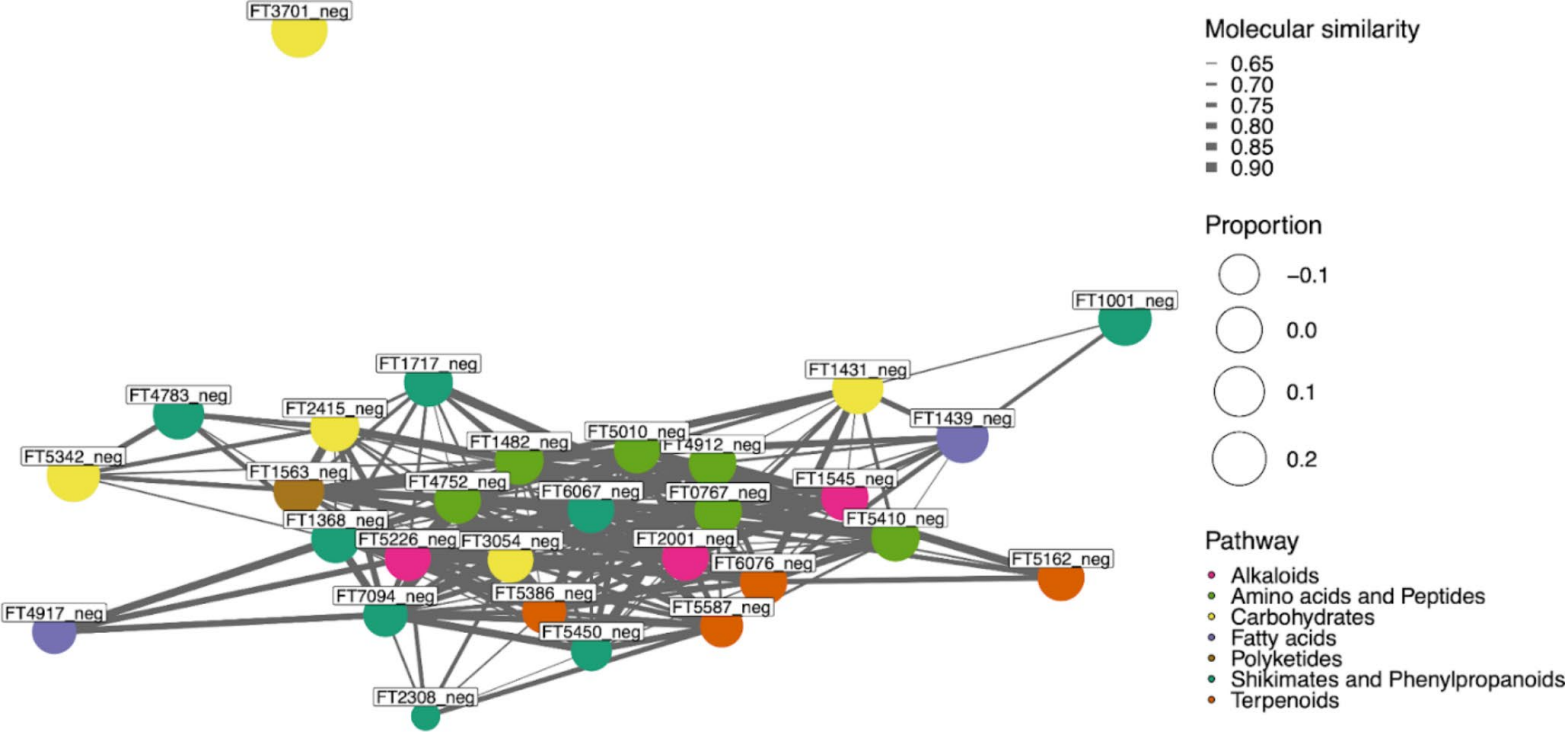
Molecular network showing the relationships of the selected compounds to pathways and compound classes. More information regarding the selected compounds is available in the Supplementary Material

Out of the 91 selected features, 71 were successfully classified (Fig. 5). 45 classes were assigned with 20 belonging to primary metabolism (representing 30 features) and 16 belonging to specialized metabolism (representing 27 features). The remaining eight classes were too broad to be constrained to a specific type of metabolism (representing 14 features). The most detected primary metabolic classes were peptides, amino acids, fatty acids, carboxylic acid derivatives and carbohydrates/carbohydrate conjugates. At the superclass level (Djoumbou Feunang et al., 2016), the largest groups were the glycosylated compounds, organonitrogen compounds, and the amines. A detailed explanation of the significant feature fluctuations can be found in the Supplementary Information. The functional relations of the selected compounds were determined by calculating a molecular network. The network revealed that the selected compounds related to the hormone treatments were largely involving biochemical pathways of alkaloids, amino acids and peptides, carbohydrates, fatty acids, polyketides, shikimates and phenylpropanoids, and terpenoids (Fig. 6).

## Discussion

Exogenous phytohormone application resulted in distinct metabolic shifts and changes in the vegetative growth in *Radula complanata*. An analysis of the shifts in classified features based on the applied phytohormone is detailed below.

### Chemical characterization

Amino acids, peptides, and analogues, fatty amides, and carbohydrates and carbohydrate conjugates were found to be the most prominent superclasses identified by our analysis. Of the carbohydrate conjugates, peptides were the most commonly detected class, followed closely by amino acids and derivatives. N-acyl amines comprised the largest group of fatty amides identified. Overall, comprehensive proteomic studies of liverworts are lacking, potentially due to the interference of liverwort secondary metabolites that have been found to interfere with protein isolation (Yadav et al., 2020). Glycosylated compounds were the most prominent carbohydrate conjugate, as well as monosaccharides and aminosaccharides. Previous work with *Plagiochila asplenioides* (L.) Dumort has identified volemitol (a sugar alcohol), sucrose (a glycosyl compound), and starch (an oligosaccharide) as major photosynthetic products (Suleiman & Lewis, 1980). Xyloglucan (an oligosaccharide), uronic acids (sugar acids and derivatives), mannose (a monosaccharide), and mixed-linkage glucans (an oligosaccharide) were all found in bryophyte cell walls (Popper & Fry, 2003) while deposition of callose (a monosaccharides) was reported to occur with pathogen infection in *Physcomitrium patens* (Hedw.) Mitt (Oliver et al., 2009).

The greater part of the variation related to chemodiversity was explained by specialized metabolites (Supplementary Information). We found significantly fewer metabolic features with less structural dissimilarity in NAA10 and slightly more features with higher dissimilarity in BAP10, and the controls G and S. Thus, hormone treatments only slightly affected the metabolism of *R. complanata* at a global level, and homeostasis is only slightly affected (except in NAA10). This can be attributed to our analytical platform which is effective at detecting small molecules, which are highly abundant in liverworts due to their oil bodies. Oil bodies serve as the site of synthesis and storage for the majority of these compounds and are suspected to be critical for plant defense against herbivory (He et al., 2013; Kanazawa et al., 2020a; Suire et al., 2000; Tanaka et al., 2016). In this analysis, diterpenoids were the most commonly detected class of prenol lipids, followed by retinoids and sesquiterpenoids. This is of note as typically, sesquiterpenoids are reported as one of the most prominent terpenoid classes in liverworts (Chen et al., 2018; Cuvertino-Santoni et al., 2017; Ghani et al., 2016; Ludwiczuk & Asakawa, 2019). It is possible that the in vitro conditions used for *R. complanata* cultivation in this study could have contributed to the increase in diterpenoid production compared to environmental samples. In *Radula complanata*, unclassed flavonoids, flavonoid glycosides, and hydroxyflavonoids were the most commonly detected flavonoids. Despite the fact that bryophytes possess an ancestral version of the flavonoid biosynthetic pathway (with fewer transcription factors and less gene duplication), they are still prolific producers of these metabolites (Davies et al., 2020). Interestingly, epiphytic bryophytes were found to have a higher flavonoid content than terrestrial bryophytes, which could contribute to the flavonoid diversity detected in *R. complanata* (Wang et al., 2017). Flavonoids are also involved in liverwort stress responses, which could have increased the number detected in this study (Yoshikawa et al., 2018). Bibenzyls and (bis)bibenzyls are a class of metabolites that are enriched in liverworts, specifically in *Radula* spp. (Asakawa et al., 2020; Ghani et al., 2016; Kageyama et al., 2015; Yoshikawa et al., 2018). While bibenzyl and (bis)bibenzyl do not belong to a single compound class in ClassyFire, the majority of these metabolites are classified as stilbenes which were repeatedly detected in this analysis. The full chemical ontology for bibenzyls can be found in the supplementary information (Table S2). (Bis)bibenzyl content has been reported to increase under several different stress conditions, suggesting that these metabolites may serve a role in plant defense (Kageyama et al., 2015; Yoshikawa et al., 2018). Previous work has also demonstrated that bibenzyl production varies with response to phytohormone application (Blatt-Janmaat et al., 2022).

### Metabolomic response to phytohormones

The consistent sample clustering in Fig. 5 demonstrated that there were clear patterns in the 91 features selected by the BORUTA analysis. Some features showed clear distinction between stress-response and growth treatments, while others were dependent on the specific phytohormone applied. FT1934 (a 1,2-amino alcohol) and FT4917 (unclassified) were both downregulated in growth treatments while FT1001 (4-(3-Methyl-2-butenyl)-5-phenethylbenzene-1,3-diol), FT1439 (a fatty acid ester), and FT1431 (unclassified) were upregulated in growth treatments. Fatty acids are key metabolites involved in plant growth, membrane function, and plant performance (Li et al., 2016) while bibenzyls may be involved in liverwort growth regulation. FT4783 (a flavonoid glycoside) and FT5342 (GDPhexose) were both upregulated in all stress-response treatments while FT2110 (an aralkylamine) was downregulated. An increase in flavonoids has been reported *Marchantia polymorpha* subjected to wounding stress (Yoshikawa et al., 2018) while GDP-hexoses are important precursors for several pathways, such as the production of ascorbic acid and cell wall production, both of which are involved in plant responses to abiotic stress (Tao et al., 2018). The remaining selected features shifted in response to each applied phytohormone and are discussed in detail below.

#### Stress response hormones

In the AA1 treatments, the significant downregulation of a peptide, a fatty acid, and a carbohydrate was observed. This suggests that liverwort primary metabolism is impacted by AA application. In the moss *Physcomitrella patens*, soluble sugars and carbohydrates increased in with AA treatment to prevent freezing damage (Takezawa et al., 2011). No significant changes in stilbenes were observed, which directly contrasts the increase in bibenzyl production that was observed with AA treatments in *Marchantia polymorpha* (Kageyama et al., 2015). A mixed response was observed from two glycosylated compounds with one being upregulated while the other was downregulated. In *Poliha nutans*, AA treatment upregulated some flavonoid genes and downregulated others (Liu et al., 2014; Zhang et al. 2019a, b). The downregulation of primary metabolites as a result of pathogen infection stress has been documented previously in the moss *P. patens* and is consistent with the observations in this study (Otero-Blanca et al., 2021). Interestingly, these changes did not occur in the AA10 treatment.

Liverworts are not typically reported to produce endogenous methyl jasmonate or MeJA-Ile due to the lack of biosynthetic enzymes (Han, 2017), and instead utilize dn-OPDA to activate the COI1 jasmonate receptor (Monte et al., 2019). In the MeJA10 treatments, an increase in a hexose and decreases in a peptide and a carbohydrate were observed. In *Radula complanata*, no significant changes in amino acids were observed with MeJA treatment. Primary metabolites were increased in *Physcomitrella patens* through the reinforcement of the cell wall (Oliver et al., 2009). Glycosylated compounds had a mixed response, with FT2016 showing an increase in MeJA1 and MeJA10 while FT4352 was decreased in MeJA10. For the secondary metabolites, a triterpenoid (FT5587) was downregulated at MeJA1 and an anisole was downregulated at MeJA10. Interestingly no changes in flavonoids, phenolics, or stilbenes were detected in the 91 selected features. In *P. patens*, MeJA induced the expression of PAL and CHS which are involved in flavonoid biosynthesis (Oliver et al., 2009). This was also observed in *Plagiochasma appendiculatum* and *Poliha nutans*, where transcription of genes in the flavonoid pathway was induced with MeJA treatment (Gao et al., 2015; Liu et al., 2014; W. Zhang et al. 2019a, b).

In the SA treatments, the majority of changes observed were downregulations and largely occurred at both concentrations. In the moss *Syntrichia ruralis*, protein content was found to decrease with SA treatment (Ruchika & Péli, 2020), which was also observed in this study. In *Physcomitrella patens*, only one SA receptive gene was identified and the pathway was found to activate following pathogen infection (Peng et al., 2017). Interestingly, transcription of a 4-coumarate CoA ligase (phenylpropanoid pathway) from *Plagiochasma appendiculatum* was found to be induced with SA treatment, suggesting that this pathway is upregulated as a response to SA (Gao et al., 2015).

#### Growth hormones

Unfortunately, there is little research present regarding the metabolomic changes as a result of applying growth promoting phytohormones to bryophytes. Previous work has identified that physical changes in the growth and maturity of bryophytes occurs with auxin and cytokinin application, however the metabolic impacts were not examined (Sabovljević et al., 2014). In the BAP treatments, fewer significant changes were observed than with other hormones. In *R. complanata*, fatty acids were increased while a hexose, a triterpenoid, and a benzenoid were all downregulated. In the NAA treatments a mix of responses was observed for a variety of metabolites. In the primary metabolism, a mixed response was observed for peptides while an increase in fatty acids and amino acids was observed. Increase in an O-glycosylated compound that was observed in our work. In *Radula complanata* the three glycosylated flavonoids that were detected did not demonstrate any significant increases with NAA treatment.

### Biochemistry

The molecular network presented in Fig. 6 identified that the hormone treatments were influencing the alkaloid, amino acid/ peptide, carbohydrate, fatty acid, polyketide, shikimates and phenylpropanoid, and terpenoid pathways. The majority of these specialized metabolite pathways are involved in the production of defense compounds and are stimulated by the stress hormone pathways. In liverworts, many of these metabolites can be found in the oil bodies which are crucial for herbivory defense (Kanazawa et al., 2020b; Labandeira et al., 2014). Many of these metabolites have also been identified as having anti-fungal activity and are produced via the MeJA and SA pathways which activate during fungal infection (Commisso et al., 2021; Matsui et al., 2019). Shifts in amino acids, peptides, and carbohydrates have also been observed as a response to physical damage in bryophytes (Y. Da Chen et al., 2021). Taken together, these results are consistent with the stress results obtained from other bryophyte species, demonstrating that this methodology is effective as identifying bryophyte stress responses.

In Sect. 3.2, a detailed breakdown of the selected metabolite fluctuations in response the applied phytohormones was presented. While it is beyond the scope of this paper, it needs to be acknowledged that these pathways do not exist in isolation and there is a constant interplay between them. In *Marchantia polymorpha*, the SA and MeJA pathway exhibited cross-talk during fungal infections, with MeJA being preferred and suppressing the activity of SA (Matsui et al., 2019). Physical changes in plant growth also result from exogenous phytohormone application, which will require metabolic changes (Sabovljević et al., 2014). Future work examining the metabolite fluctuations and changes in plant growth form could be done to further elucidate the total impact of these applied hormones.

## Conclusion

In conclusion, exogenous phytohormone application resulted in metabolic shifts in *Radula complanata*. Primary and specialized metabolisms appeared to be equally impacted by phytohormone treatments, with 20 classes belonging to primary metabolism (representing 30 features) and 16 classes belonging to specialized metabolism (representing 27 features) tentatively identified. Stress-response hormones largely downregulated primary metabolites and increased or varied the production of specialized metabolites. By contrast, growth hormones largely upregulated or varied the production of primary metabolites and varied or downregulated stress-response metabolites. 4-(3-Methyl-2-butenyl)-5-phenethylbenzene-1,3-diol was identified as a biomarker for the growth treatments while GDP-hexose was identified as a biomarker for the stress-response treatments. The majority of these observations deviated from results obtained for vascular plants, highlighting the unique metabolic processes of liverworts. Future work identifying key biomarkers that are unique to liverwort metabolism could be conducted to provide more insight into liverwort phytochemistry. Our chosen approach using untargeted LC/MS revealed results that can be useful in subsequent studies and for hypothesis generation. To identify the mechanistic components of metabolic stress in the liverwort *R. complanata*, more elaborate analytical analyses are necessary to identify compounds and to determine the relationships within pathways and metabolic networks (Nothias et al., 2020), i.e., targeted LC/MS, or NMR.

## Electronic supplementary material

Below is the link to the electronic supplementary material.

Supplementary Material 1

Supplementary Material 2

Supplementary Material 3

## Data Availability

The metabolomics and metadata reported in this paper have been deposited to MetaboLights (https://www.ebi.ac.uk/metabolights/) with the identifiers MTBLS4321 and MTBLS3563. Code to reproduce the results and the figures in this study are available on Zenodo (https://zenodo.org/record/7581479, doi:10.5281/zenodo.7581479).
