# Supplementary material for: Impact of in vitro phytohormone treatments on the metabolome of the leafy liverwort Radula complanata (L.) Dumort
Source: Metabolomics. 2023 Mar 9;19(3):17. doi: 10.1007/s11306-023-01979-y (PMC9998581; doi:10.1007/s11306-023-01979-y)
Supplement: Supplementary file 1 — Supplementary Material 1 [file 11306_2023_1979_MOESM1_ESM.docx]

**Supplementary Information**: Impact of *in vitro* hormone treatments on the metabolome of *Radula* *complanata*

- 1. **Extracted Ion Chromatograms (EICs)**

To validate the chromatographic runs and peak detection, nine reference compounds were monitored throughout the runs to assess instrument drift and peak shape.

**Table S1.** LC data for reference compounds.

| **Name** | **Formula** | **Exact mass** | **RT (min)** |
| --- | --- | --- | --- |
| 4-prenyldihydropinosylvin | C_19_H_22_O_2_ | 282.16203 | 10.3 |
| Radulannin L | C_19_H_20_O_3_ | 296.14119 | 9.88 |
| Radulannin A | [C_19_H_20_O_2_](https://pubchem.ncbi.nlm.nih.gov/#query=C19H20O2) | 280.14628 | 11.21 |
| Radulannin H | C_20_H_20_O_2_ | 324.13616 | 11.32 |
| 2-(3,7-Dimethylocta-2,6-dienyl)-5-(2-phenylethyl)benzene-1,3-diol | C_24_H_30_O_2_ | 350.22426 | 12.62 |
| 3,5-dihydroxy-6-carbomethoxy-2-(3-methyl-2-butenyl)bibenzyl | C_21_H_24_O_4_ | 340.16769 | 12.46 |
| Kinetin | C_10_H_9_N_5_O | 215.08081 | 3.6 |
| Biochanin A | C_16_H_12_O_30_ | 284.06856 | 9.15 |
| N-(3-Indolylacetyl)-L-alanine | C_13_H_14_N_2_O_3_ | 246.10061 | 5.67 |


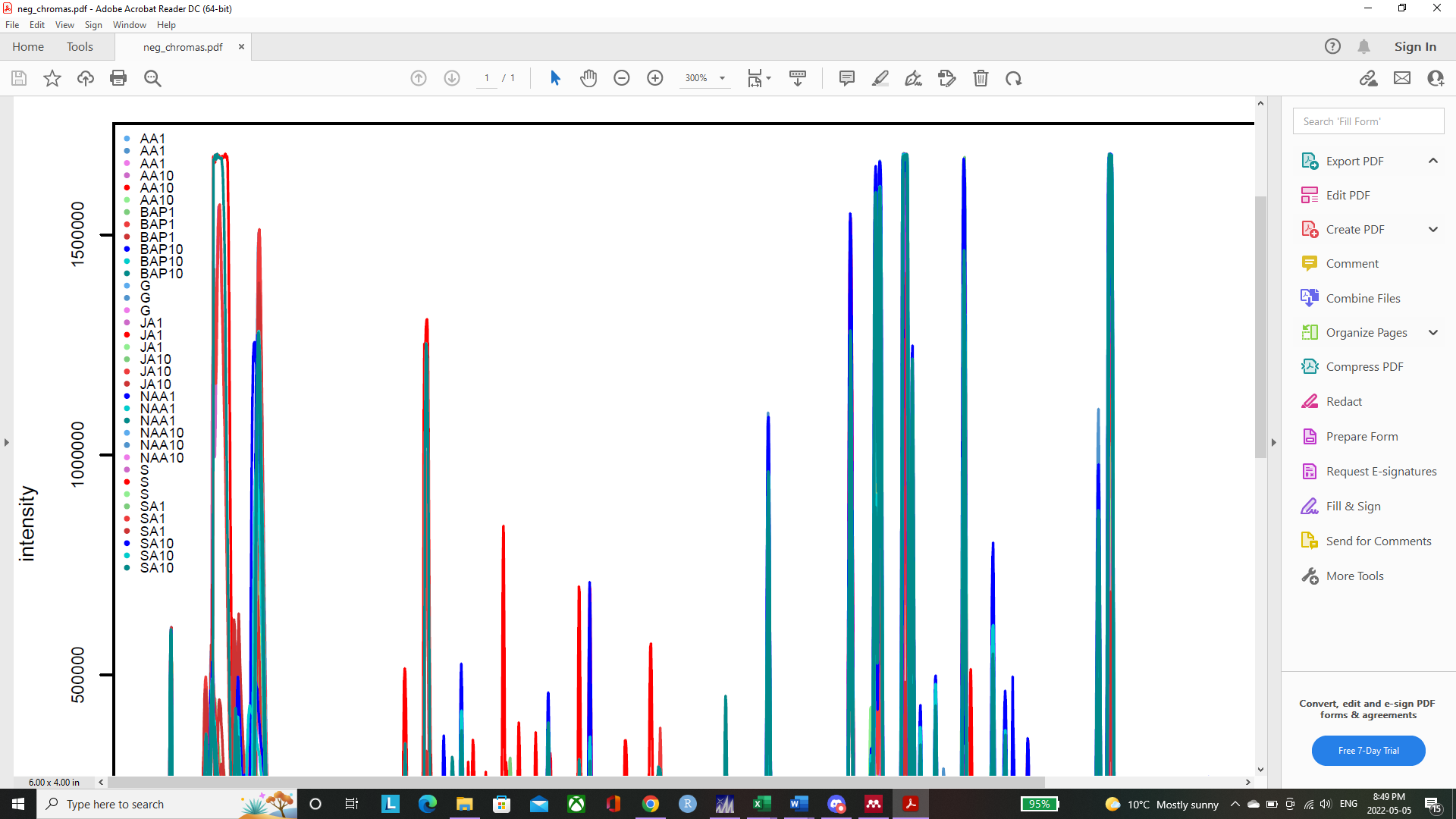
**
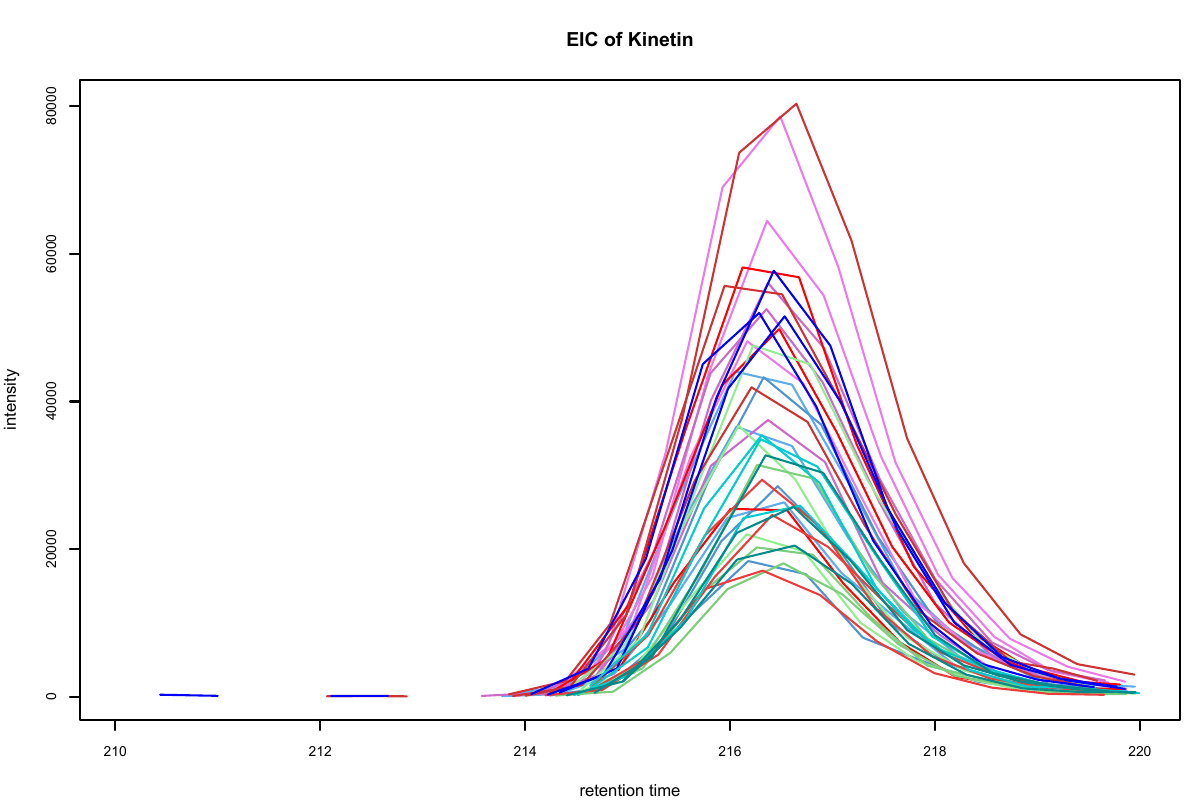
Figure S1.** EIC of Kinetin in negative mode.


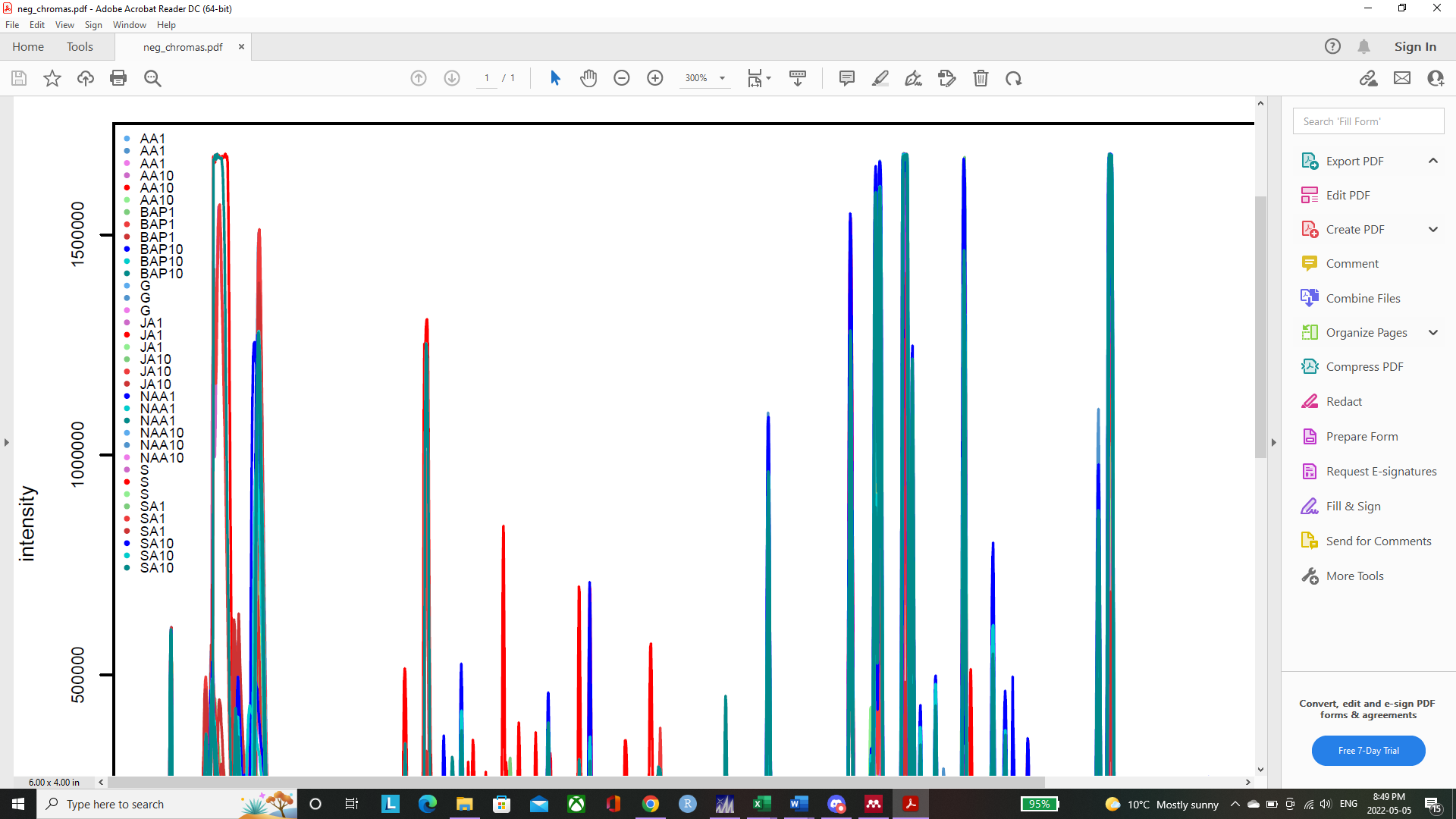
**
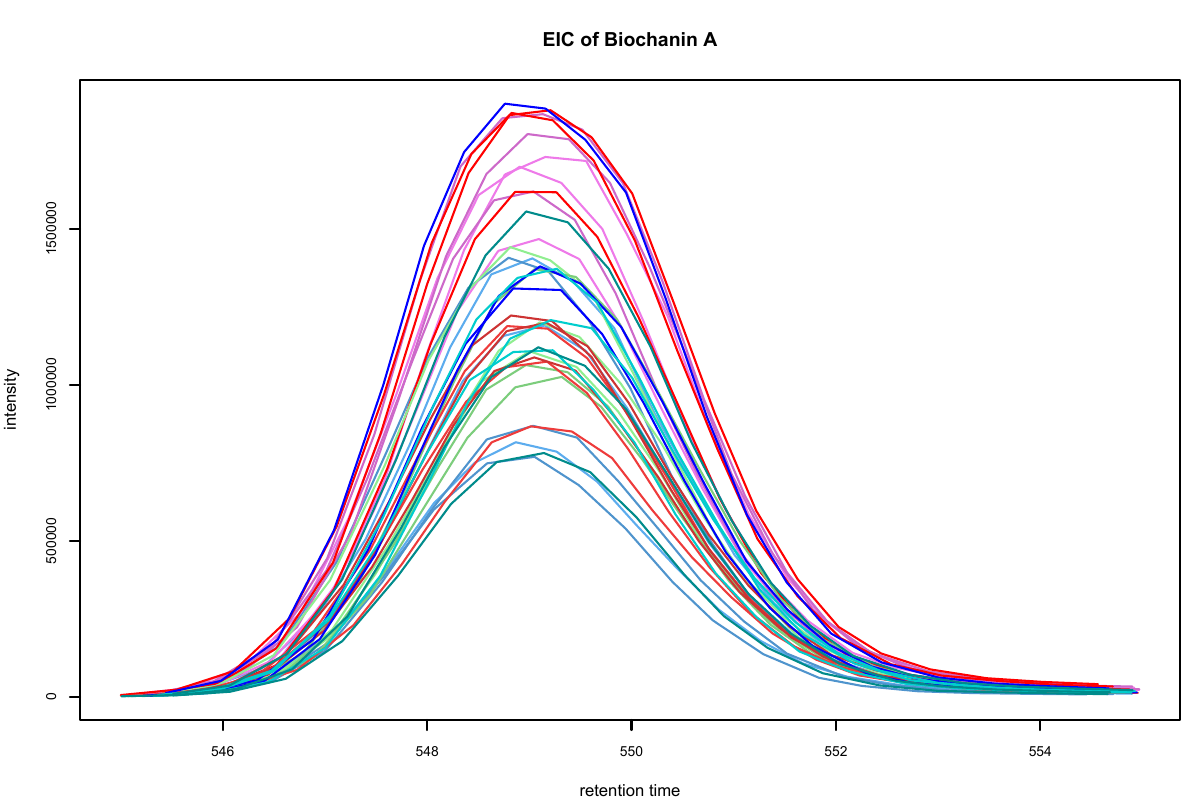
Figure S2**. EIC of Biochanin A in negative mode.


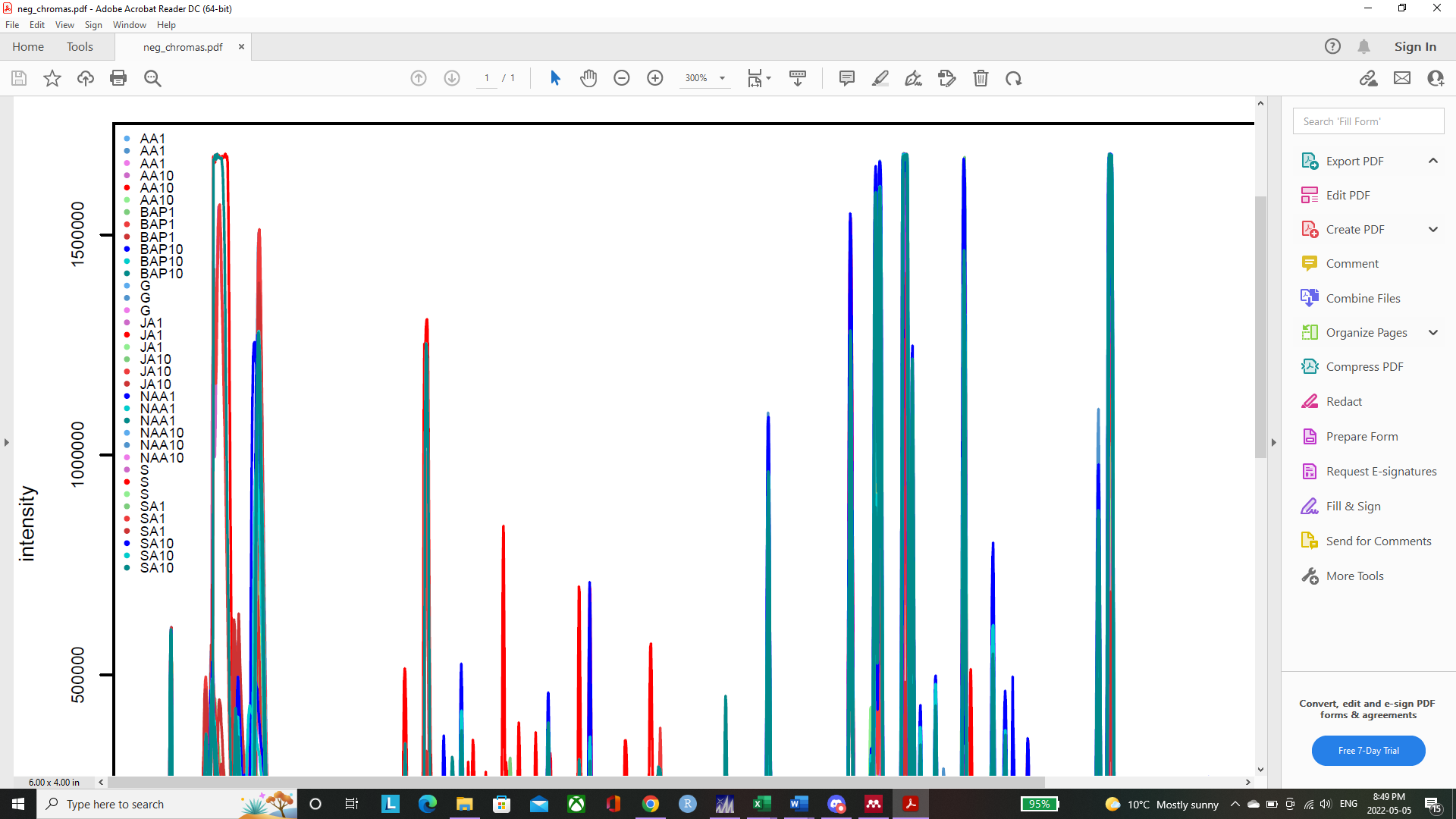
**
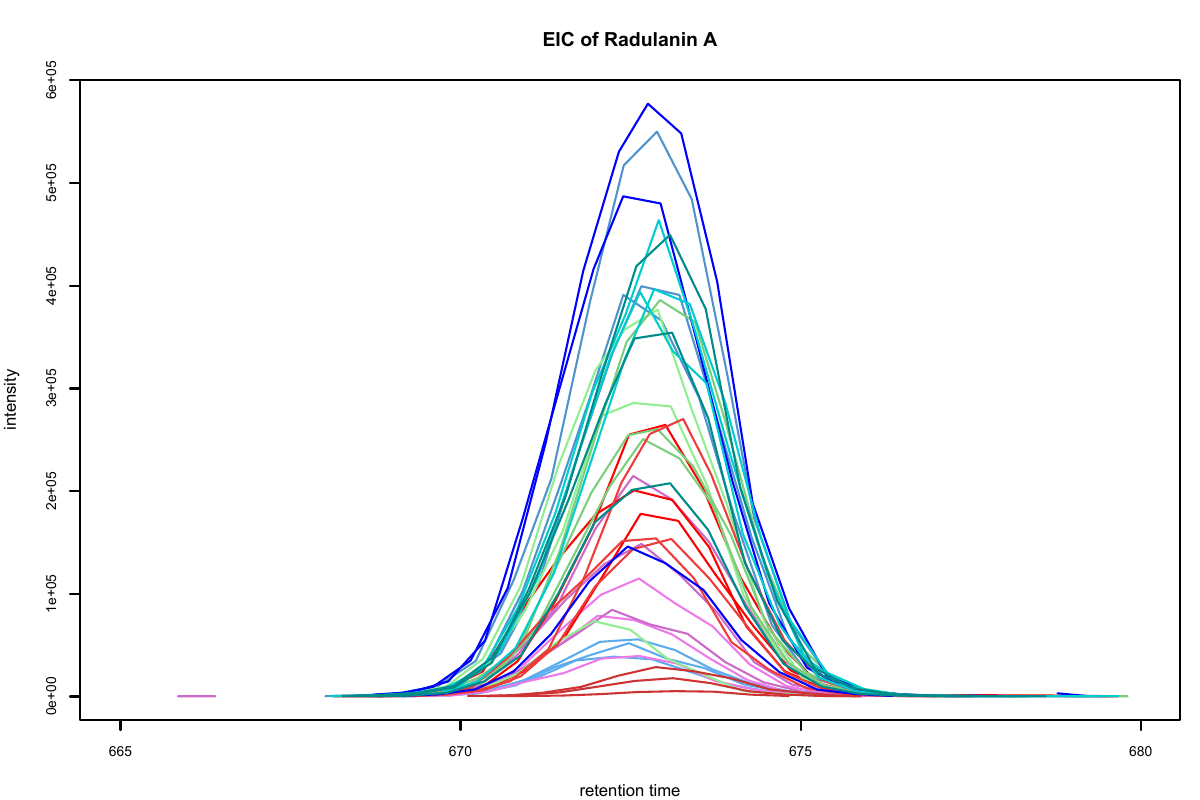
Figure S3**. EIC of Radulanin A in negative mode.


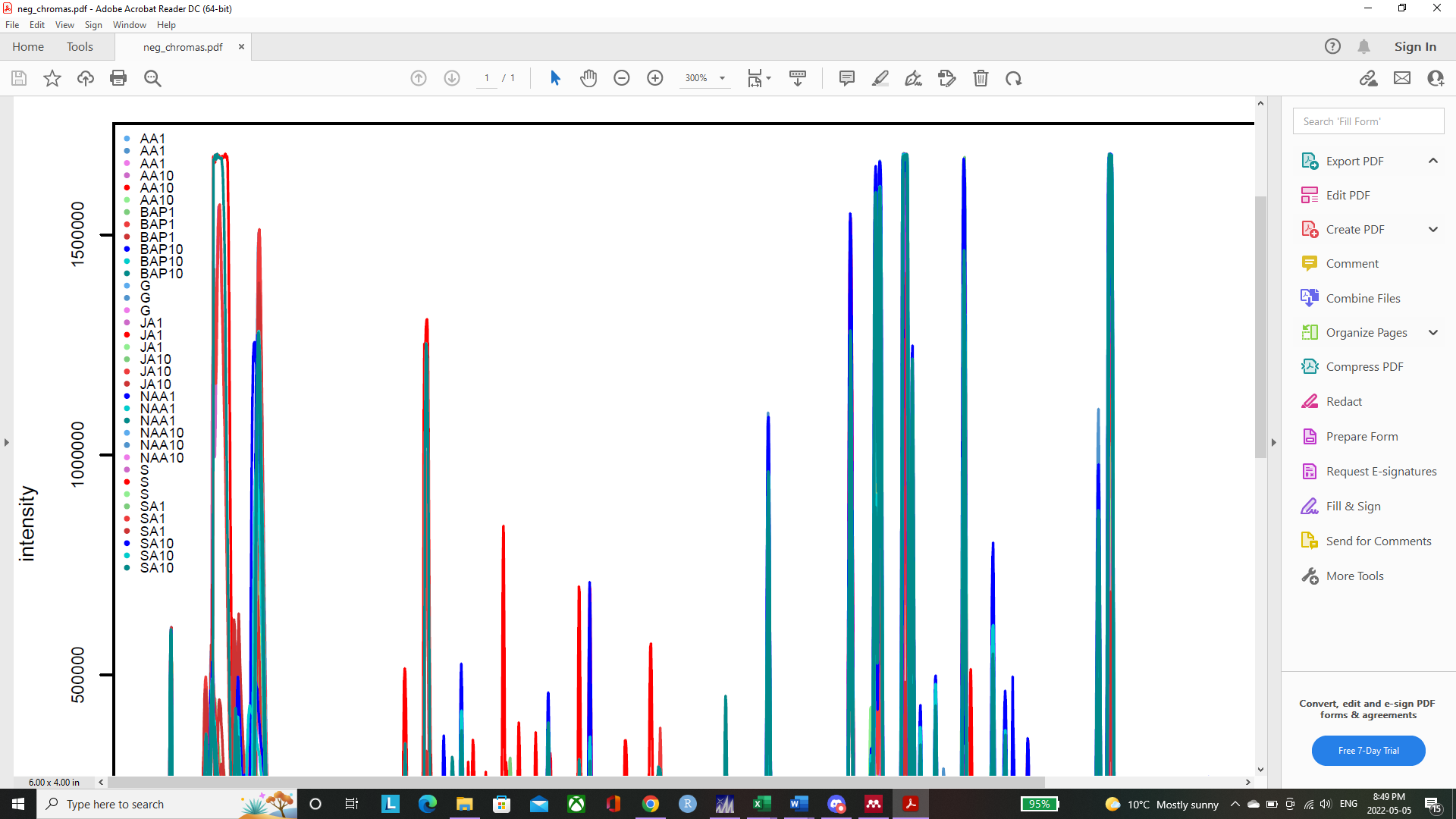
**
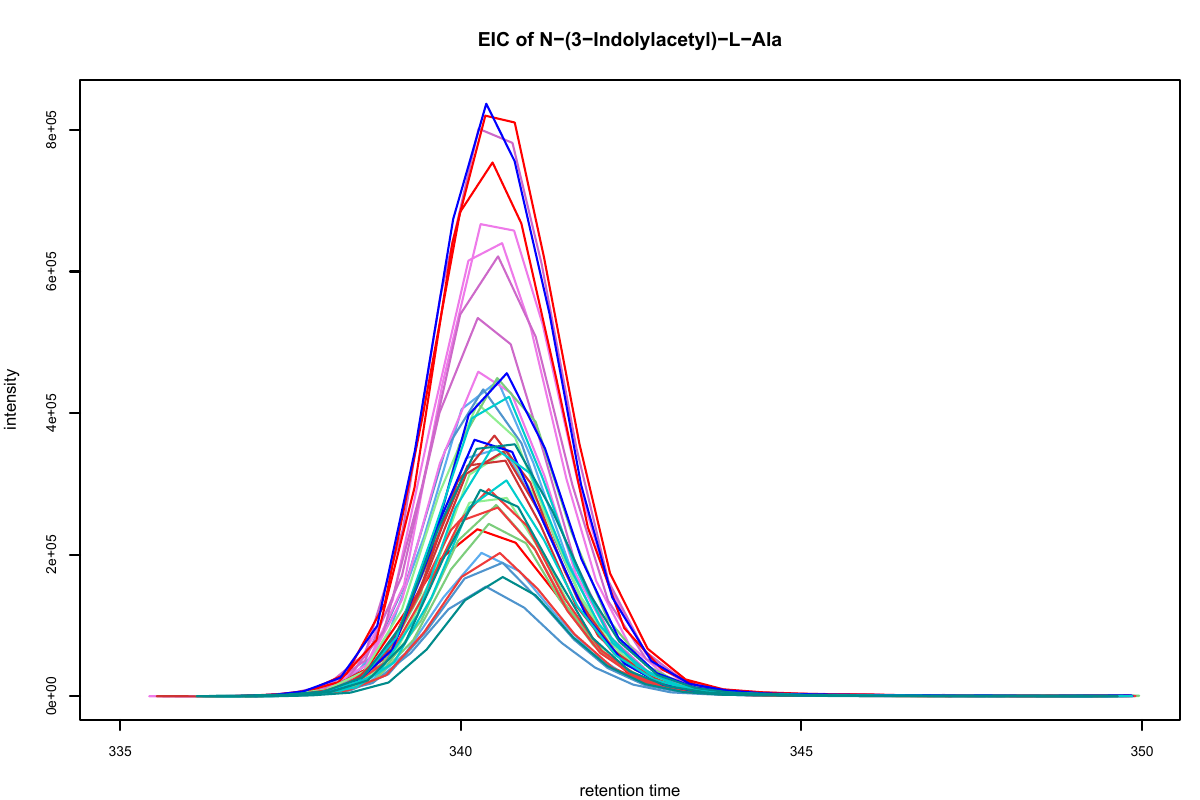
Figure S4.** EIC of N-(3-Indolylacetyl)-L-alanine in negative mode.


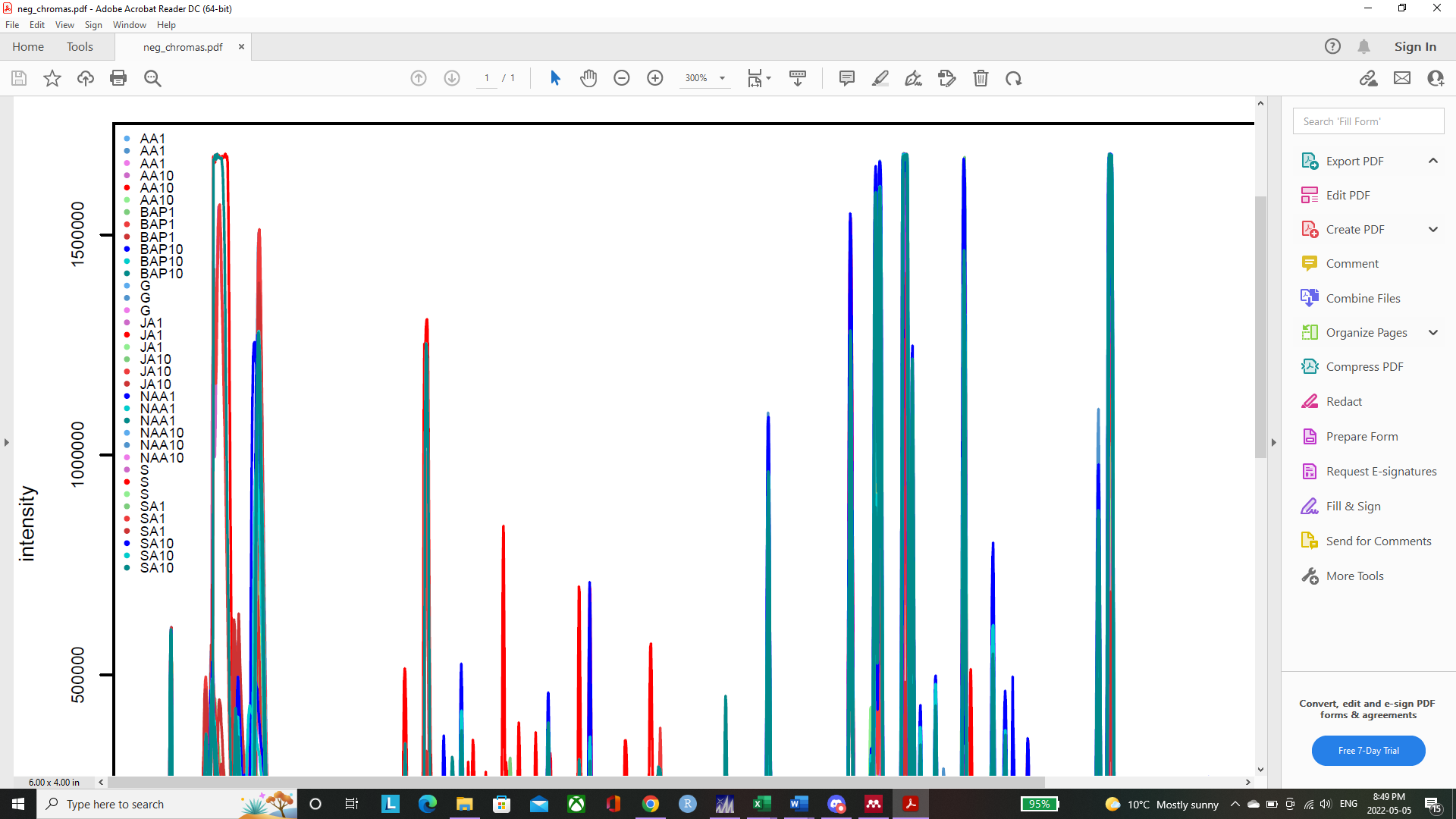
**
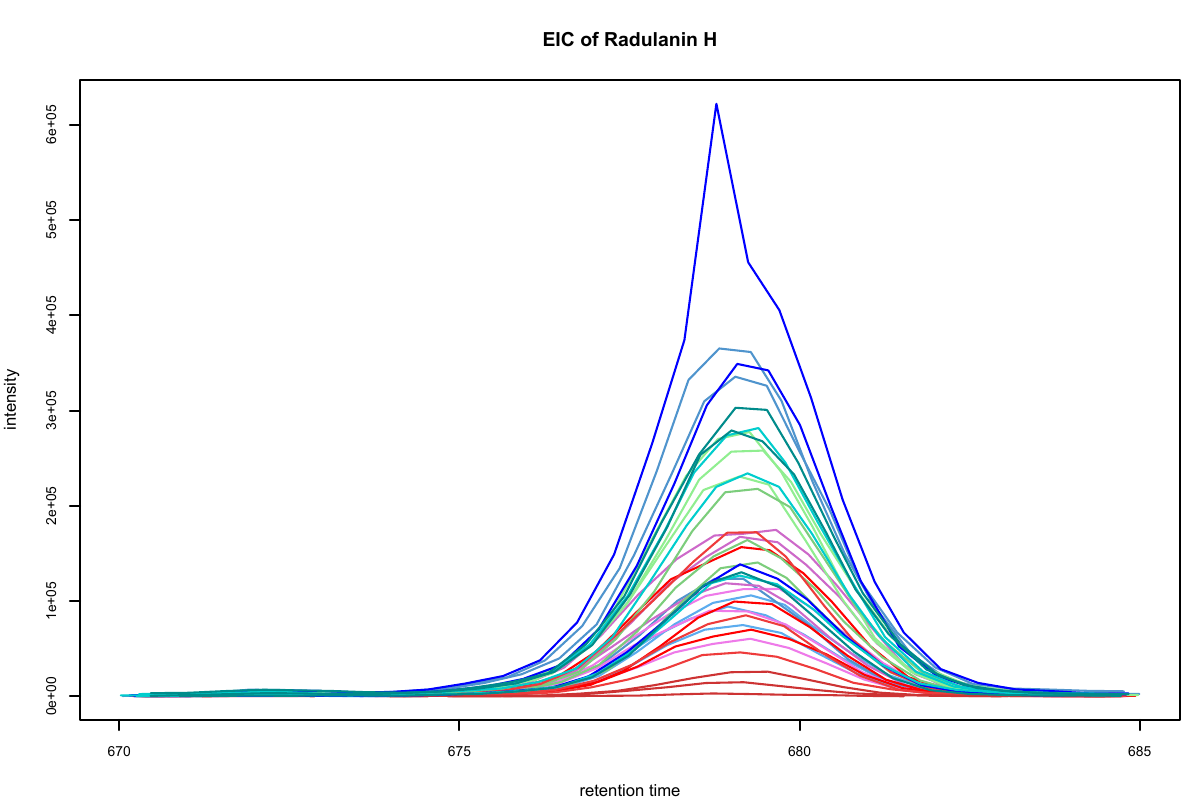
Figure S5.** EIC of Radulanin H in negative mode.


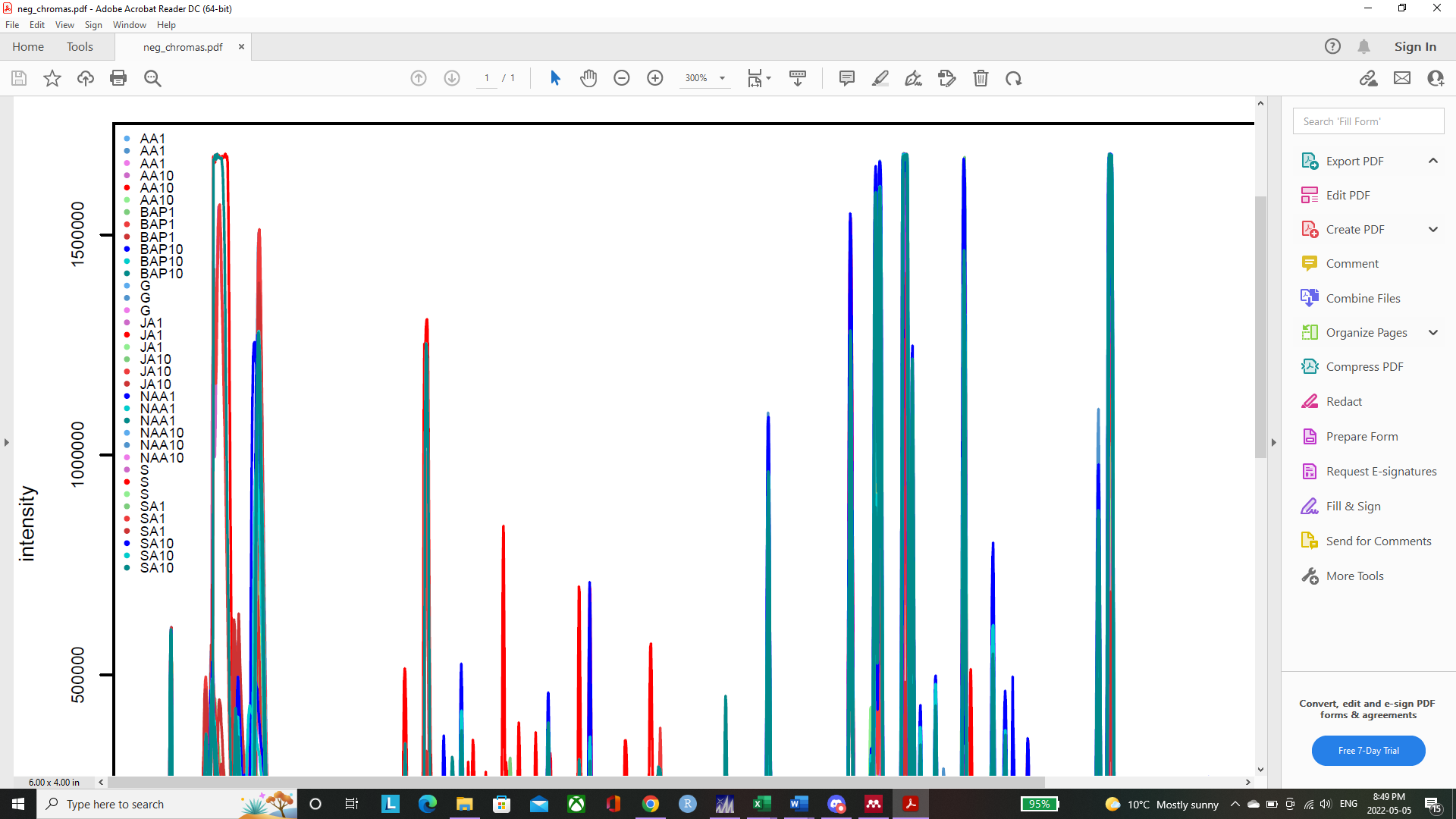
**
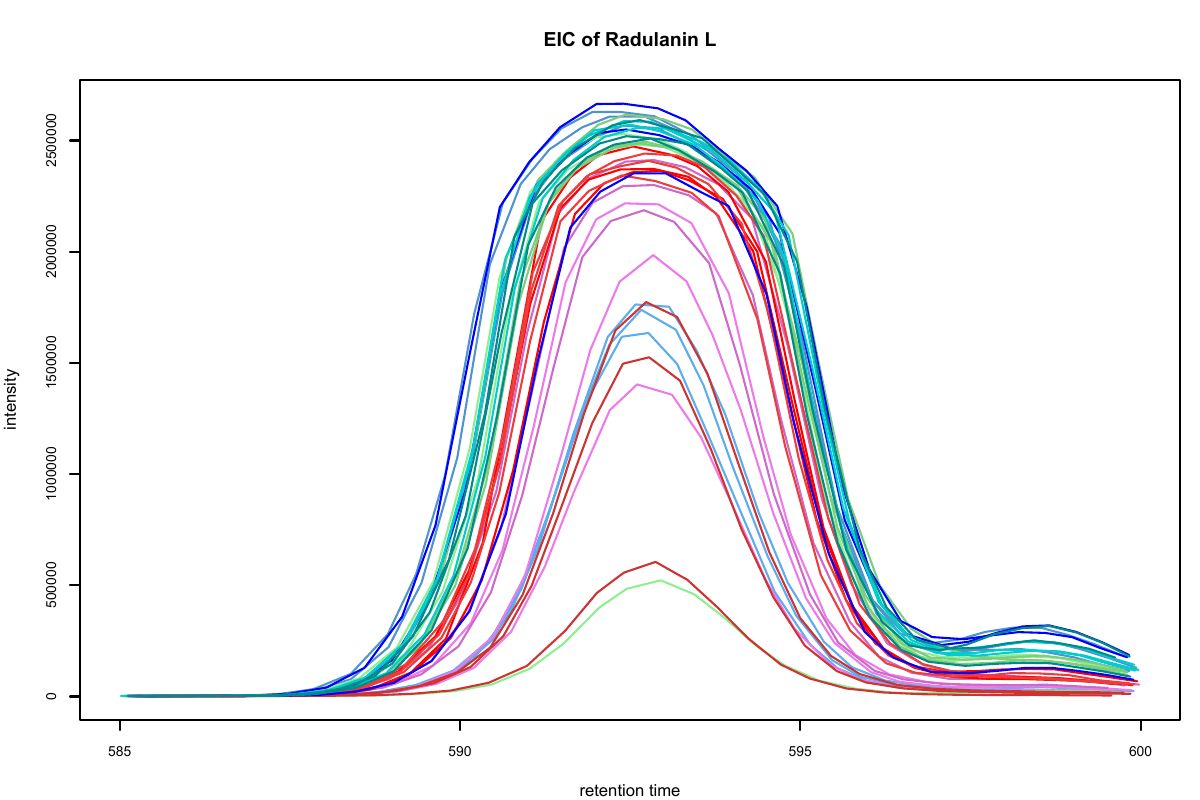
Figure S6**. EIC of Radulanin L in negative mode.


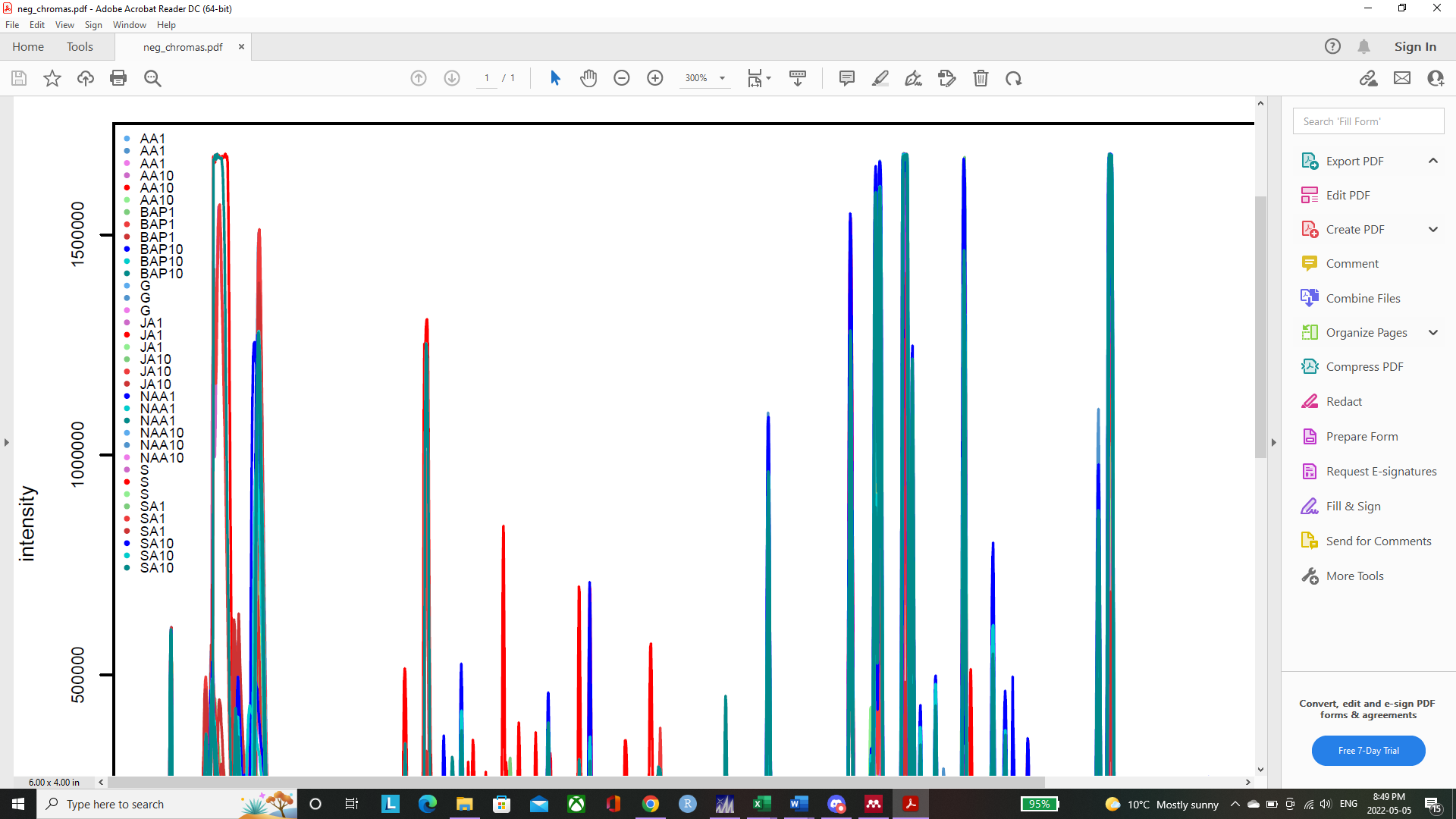

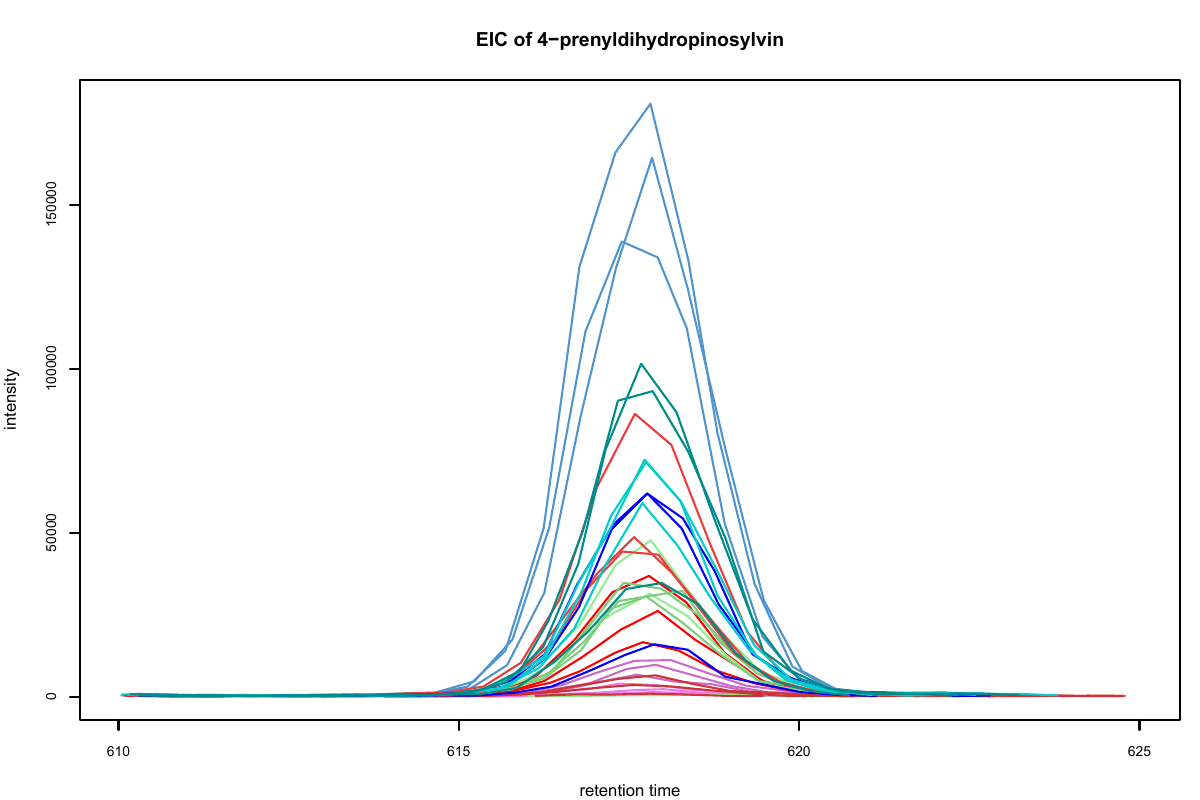
**Figure S7**. EIC of 4-prenyldihydropinosylvin in negative mode.


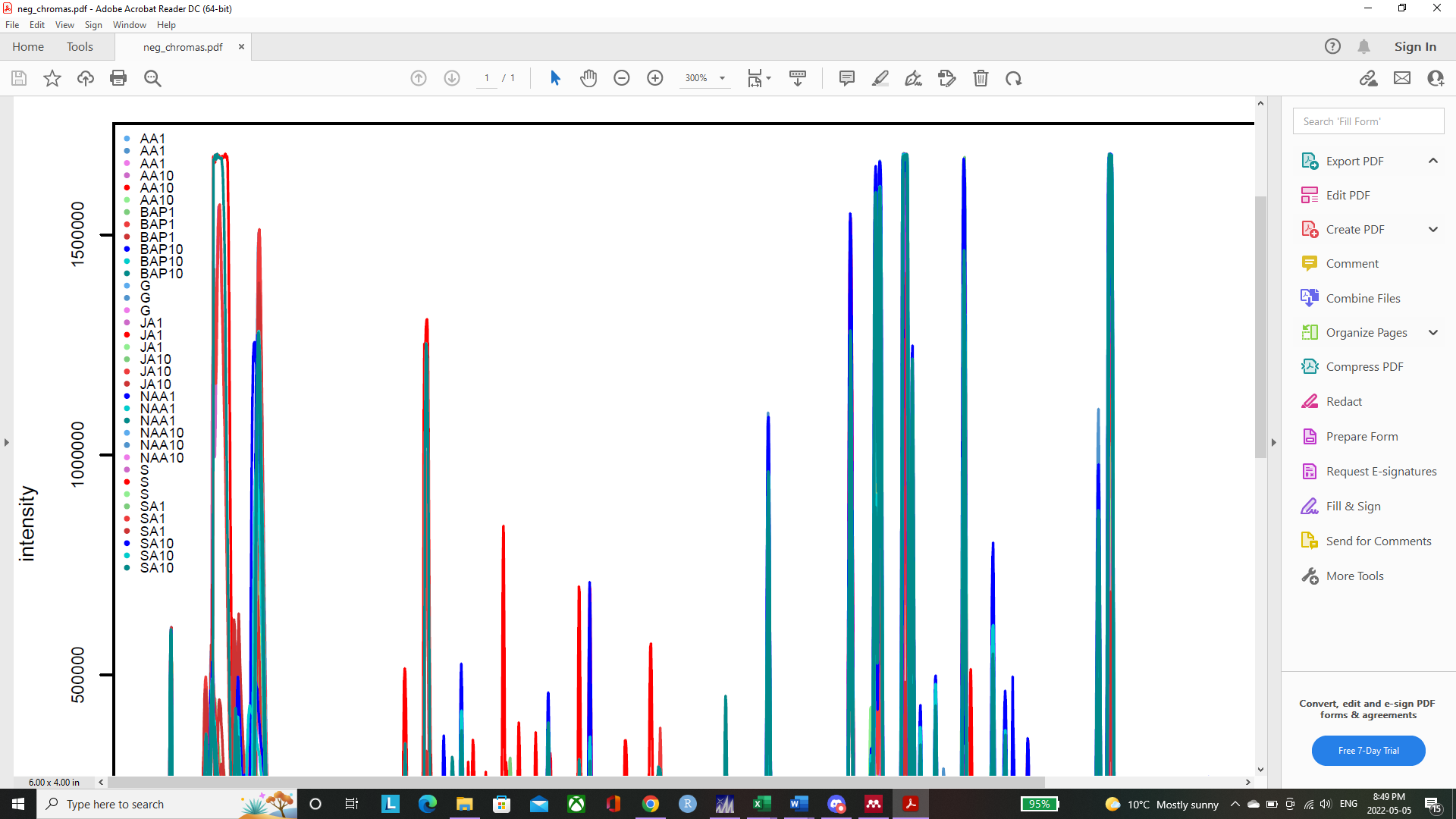

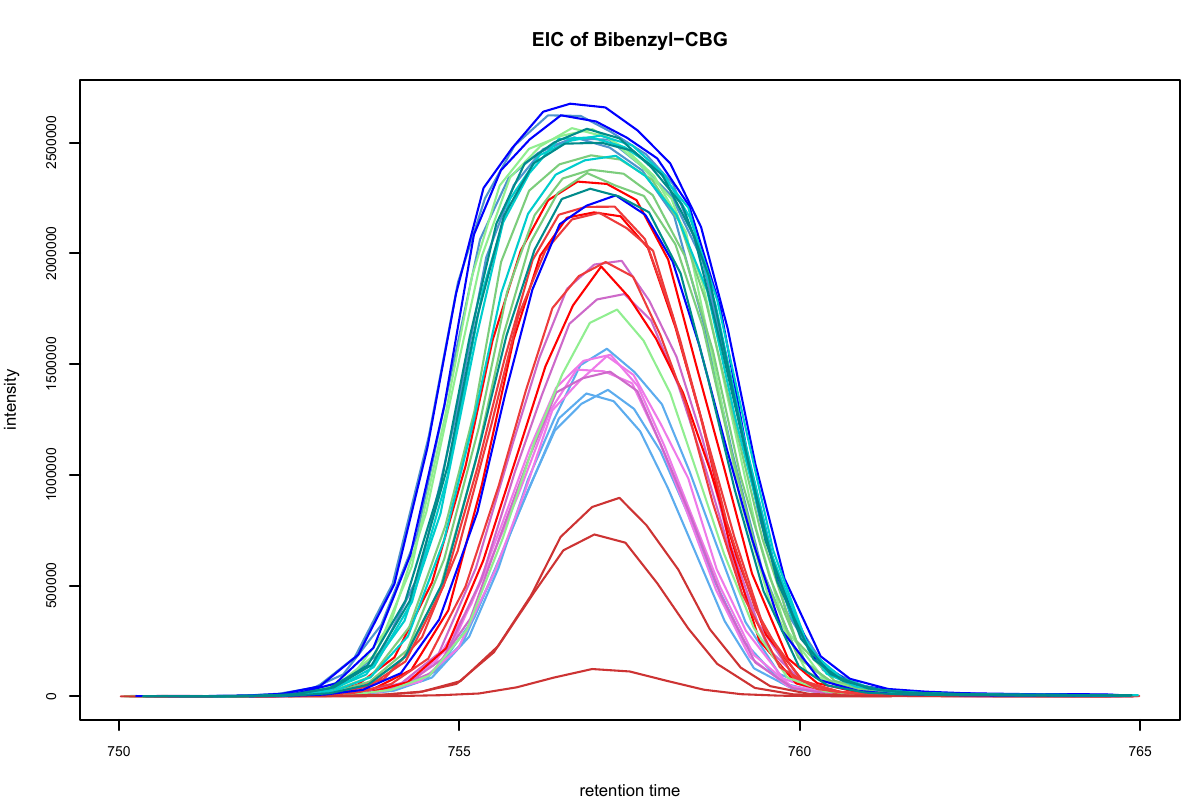
**Figure S8**. EIC of 2-(3,7-Dimethylocta-2,6-dienyl)-5-(2-phenylethyl)benzene-1,3-diol in negative mode.


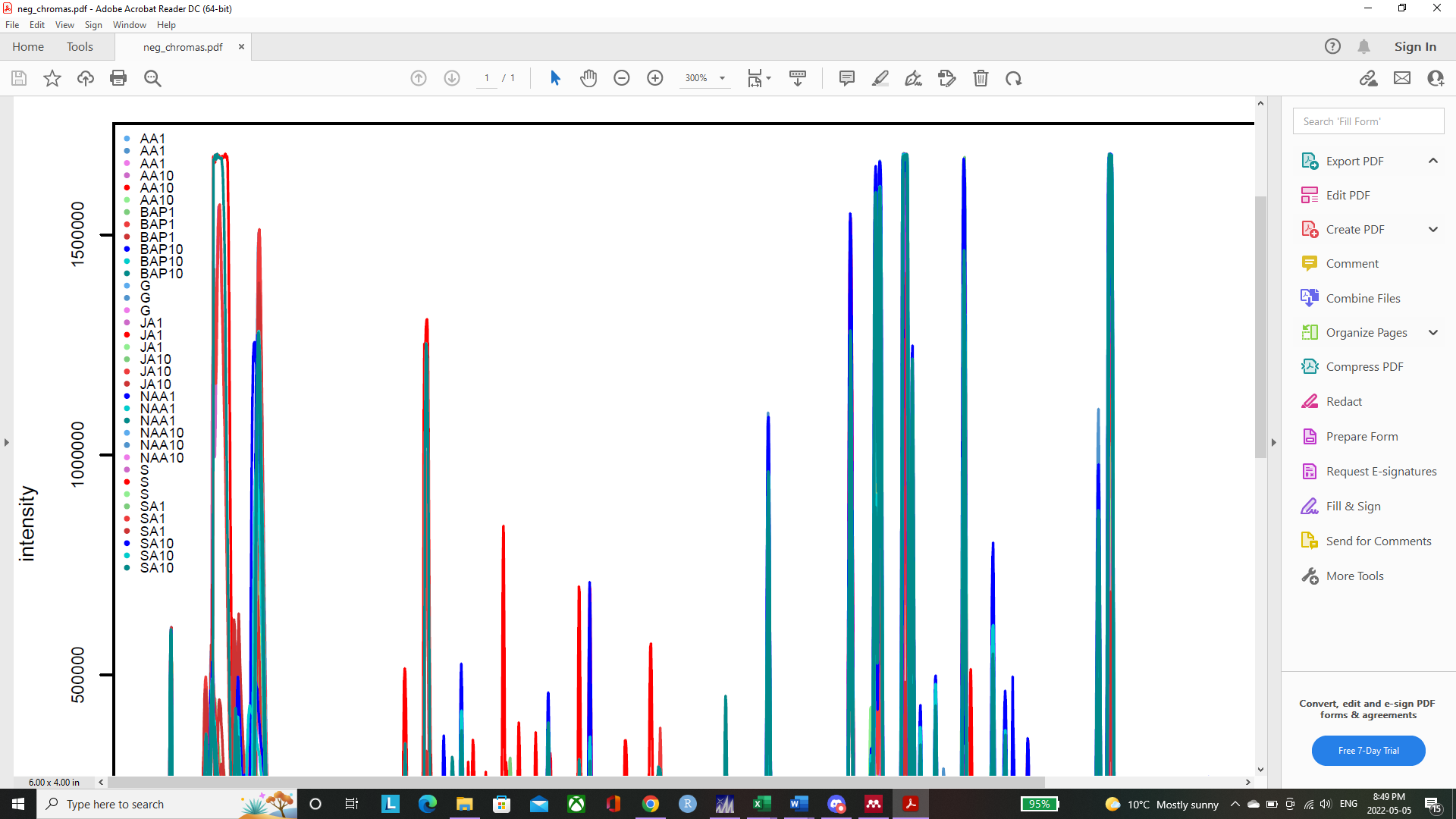

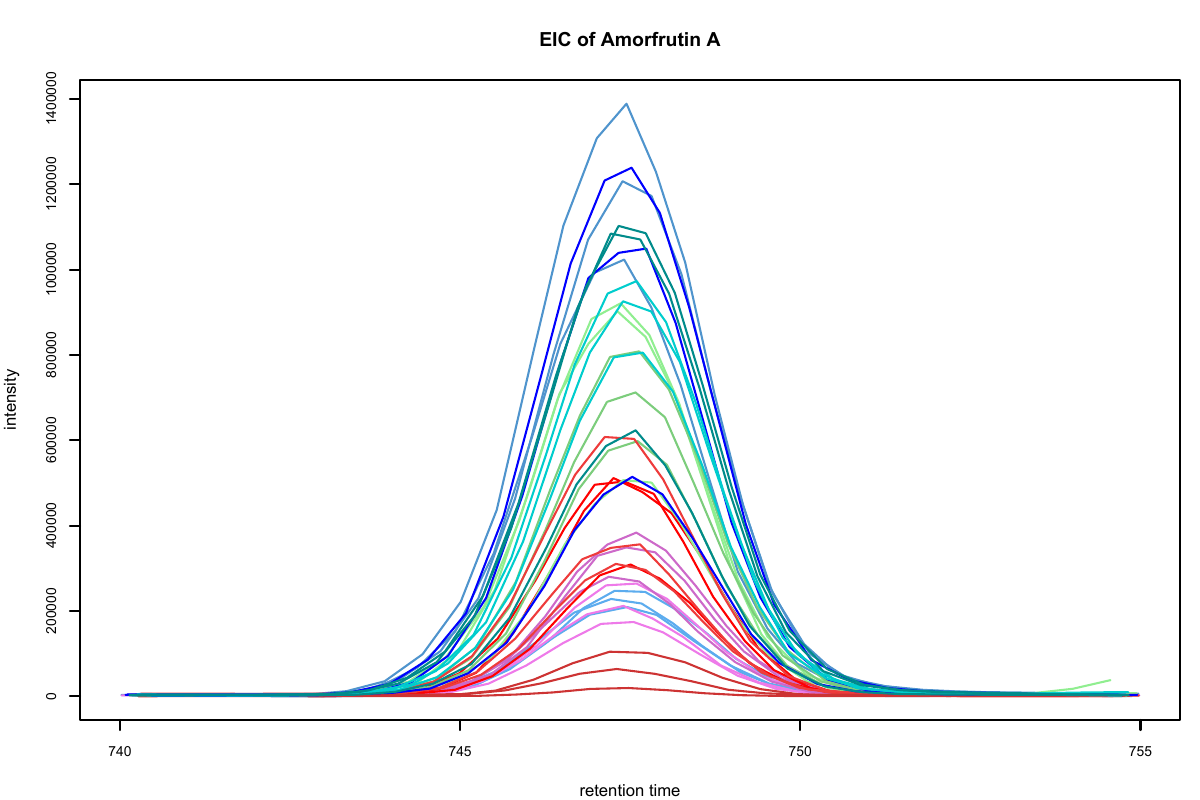
**Figure S9.** EIC of 3,5-dihydroxy-6-carbomethoxy-2-(3-methyl-2-butenyl)bibenzyl in negative mode.


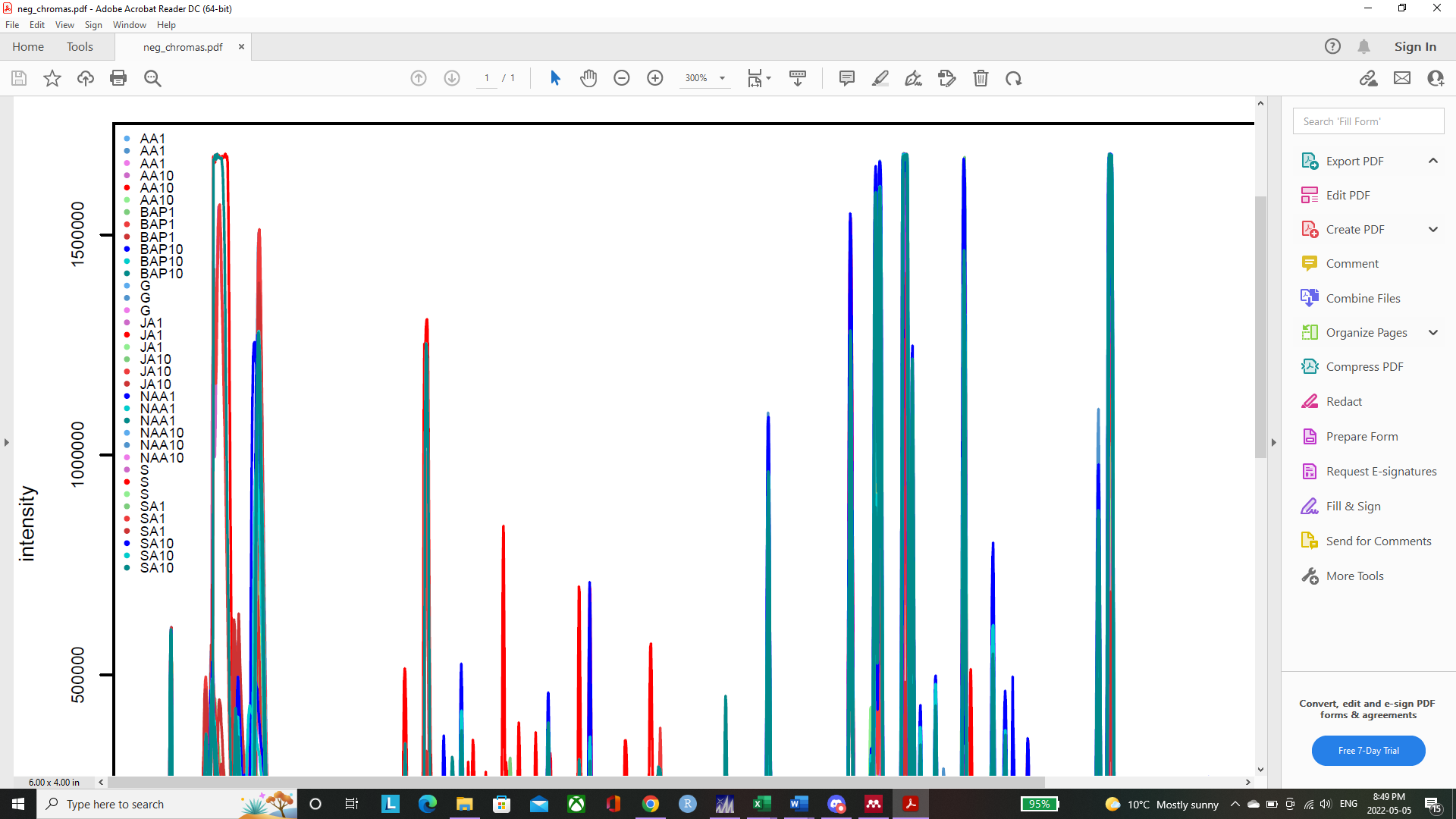
**
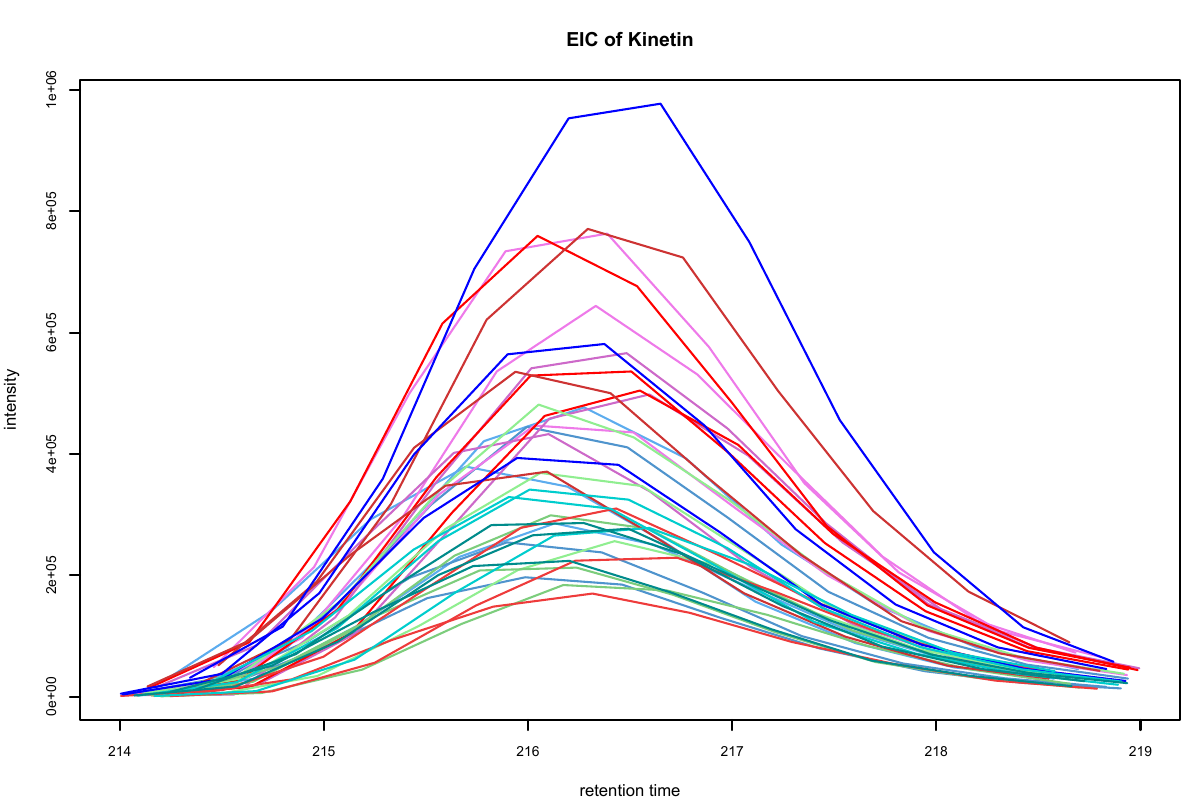
Figure S10.** EIC of Kinetin in positive mode.


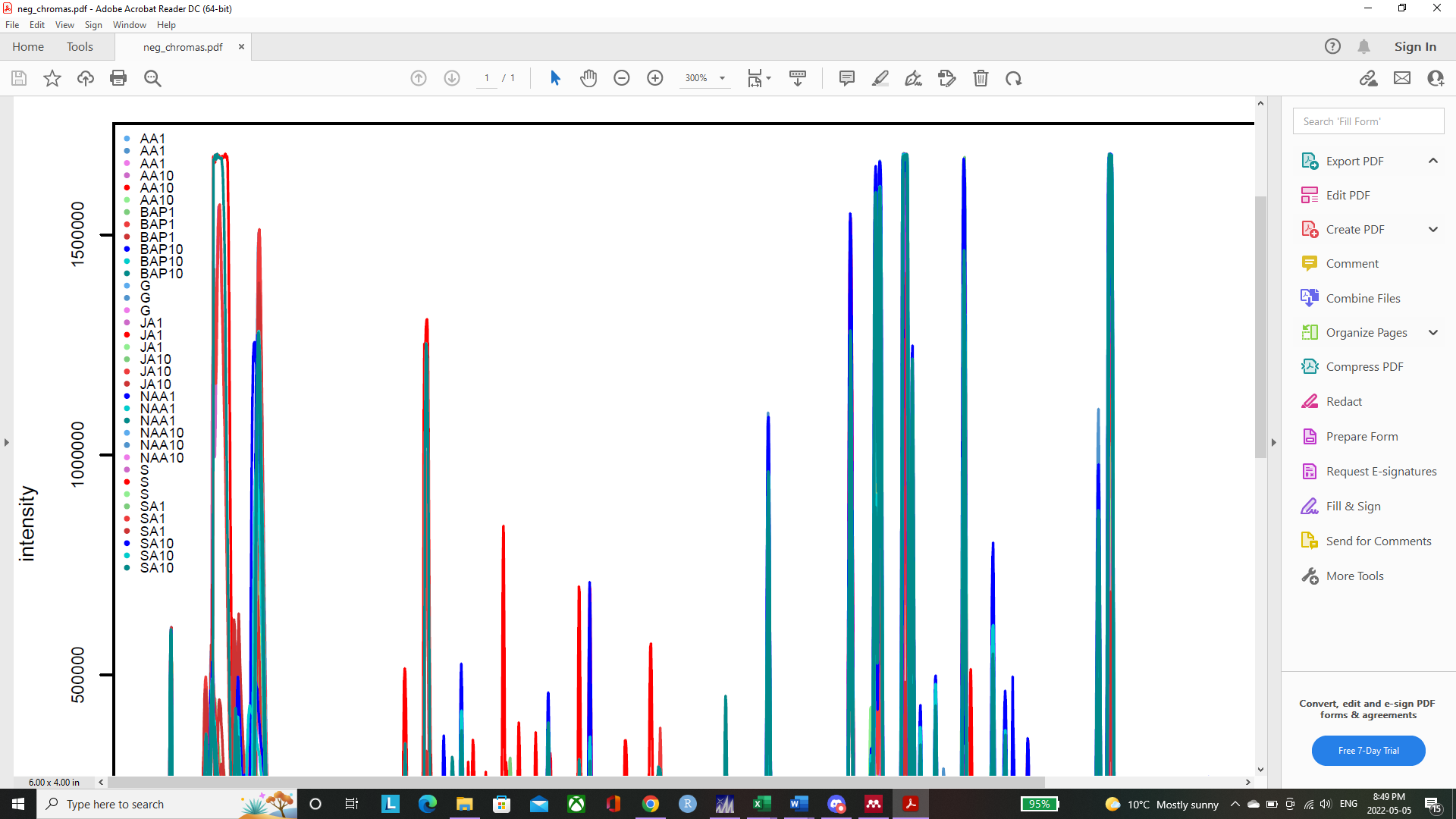
**
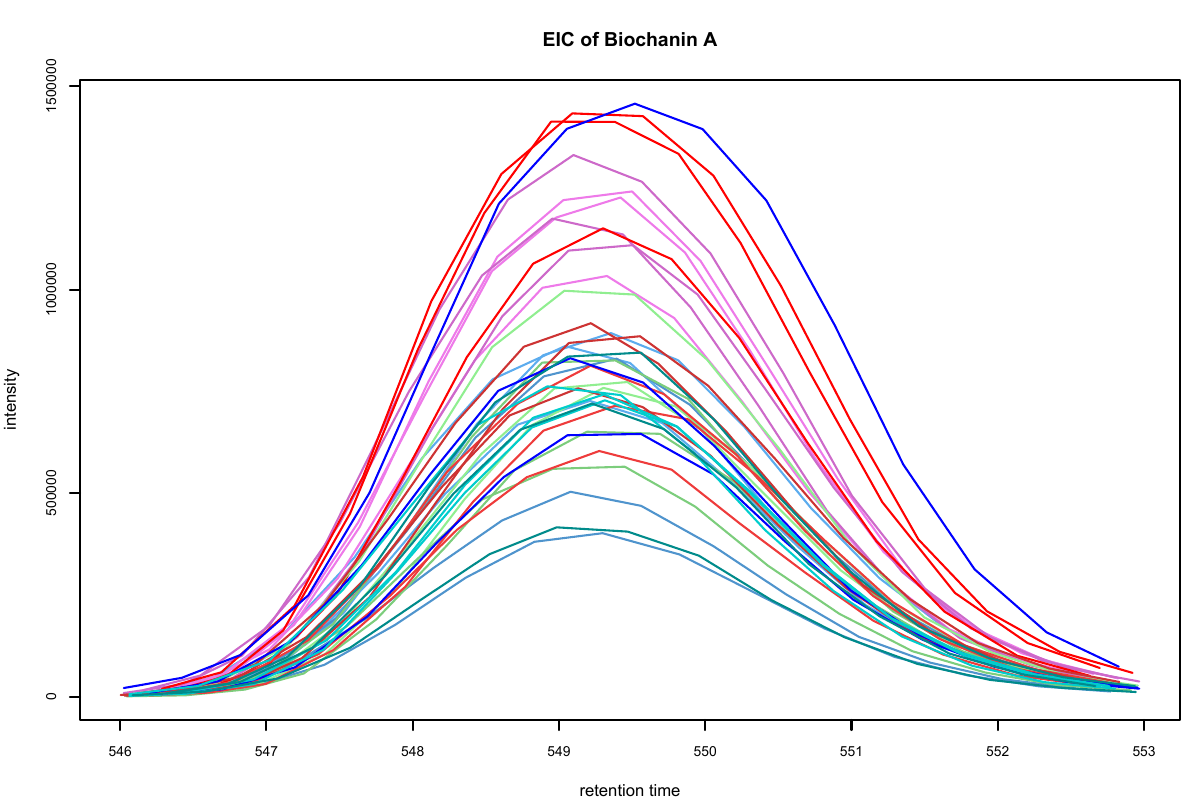
Figure S11.** EIC of Biochanin A in positive mode.


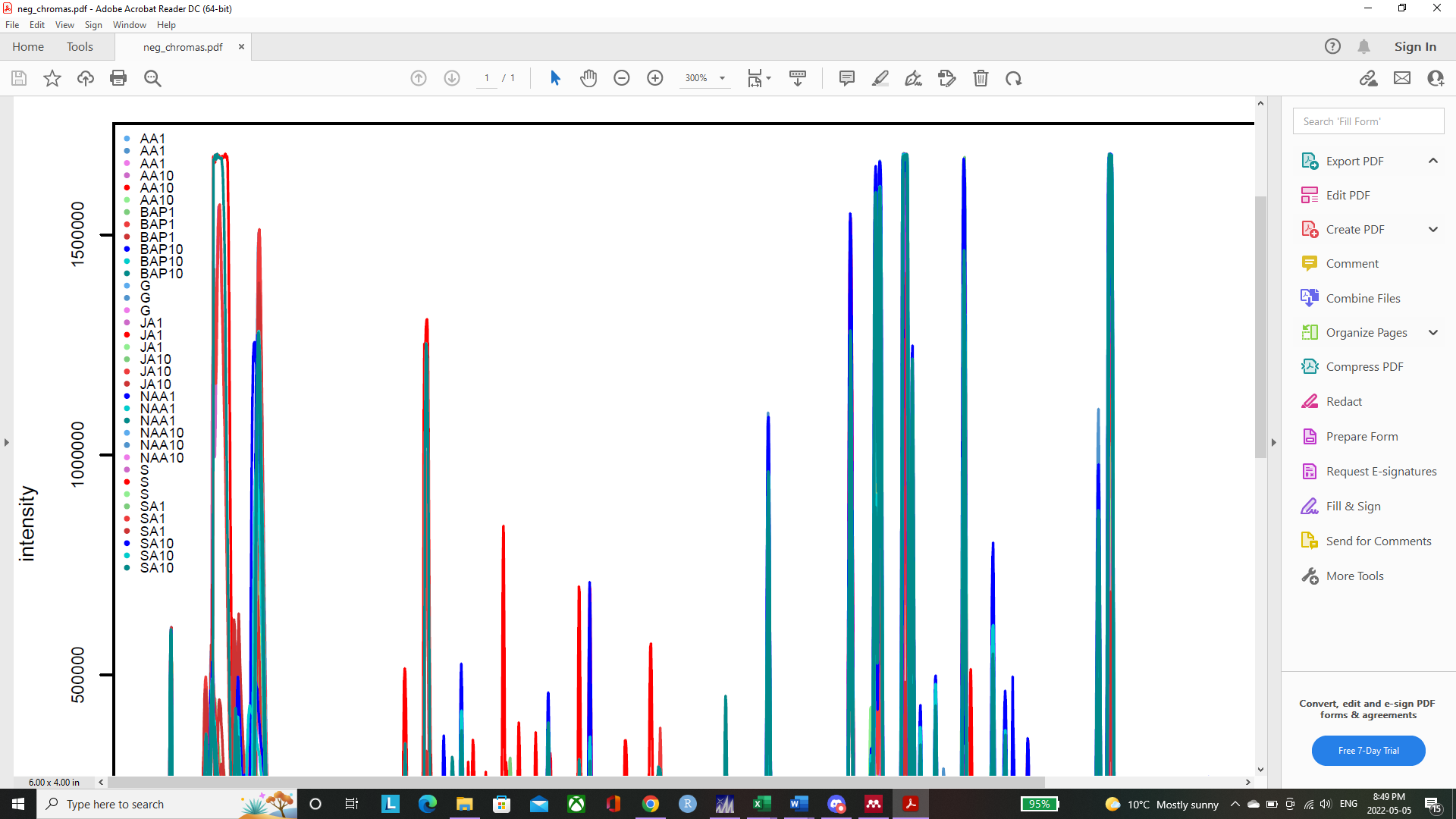
**
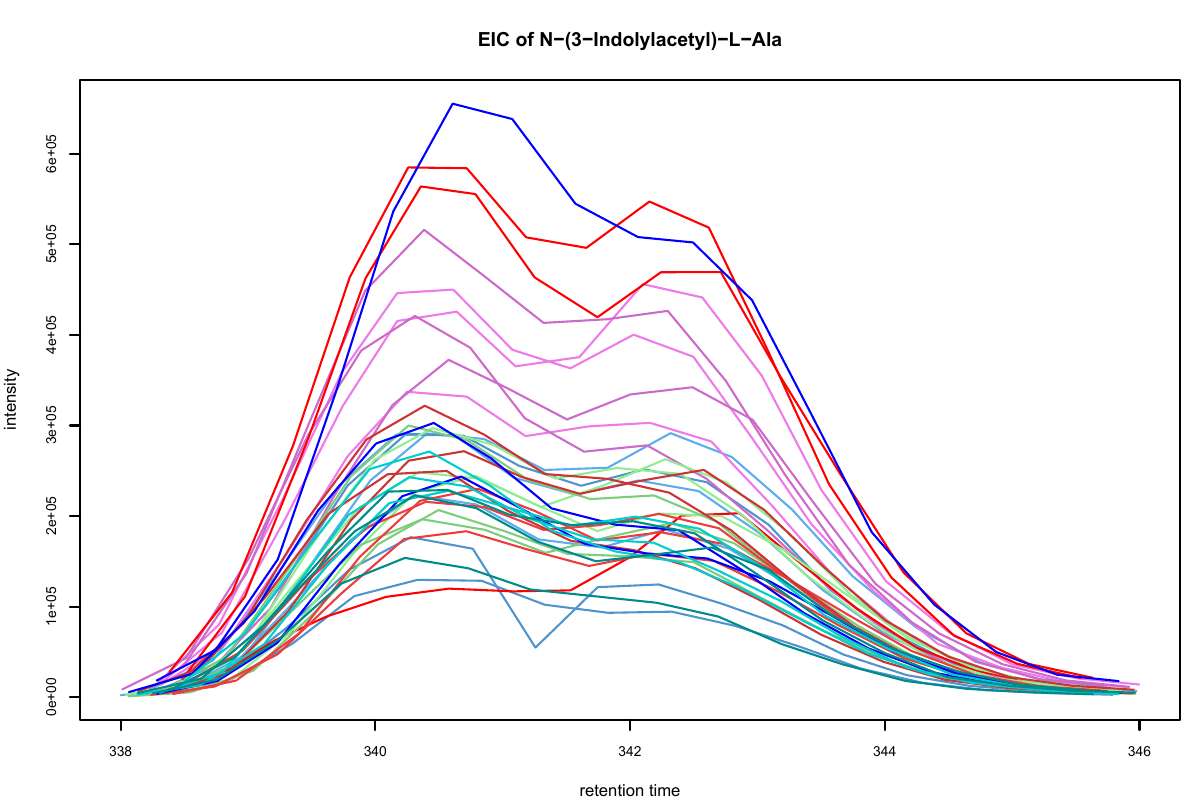
 Figure S12.** EIC of N-(3-Indolylacetyl)-L-alanine in positive mode.


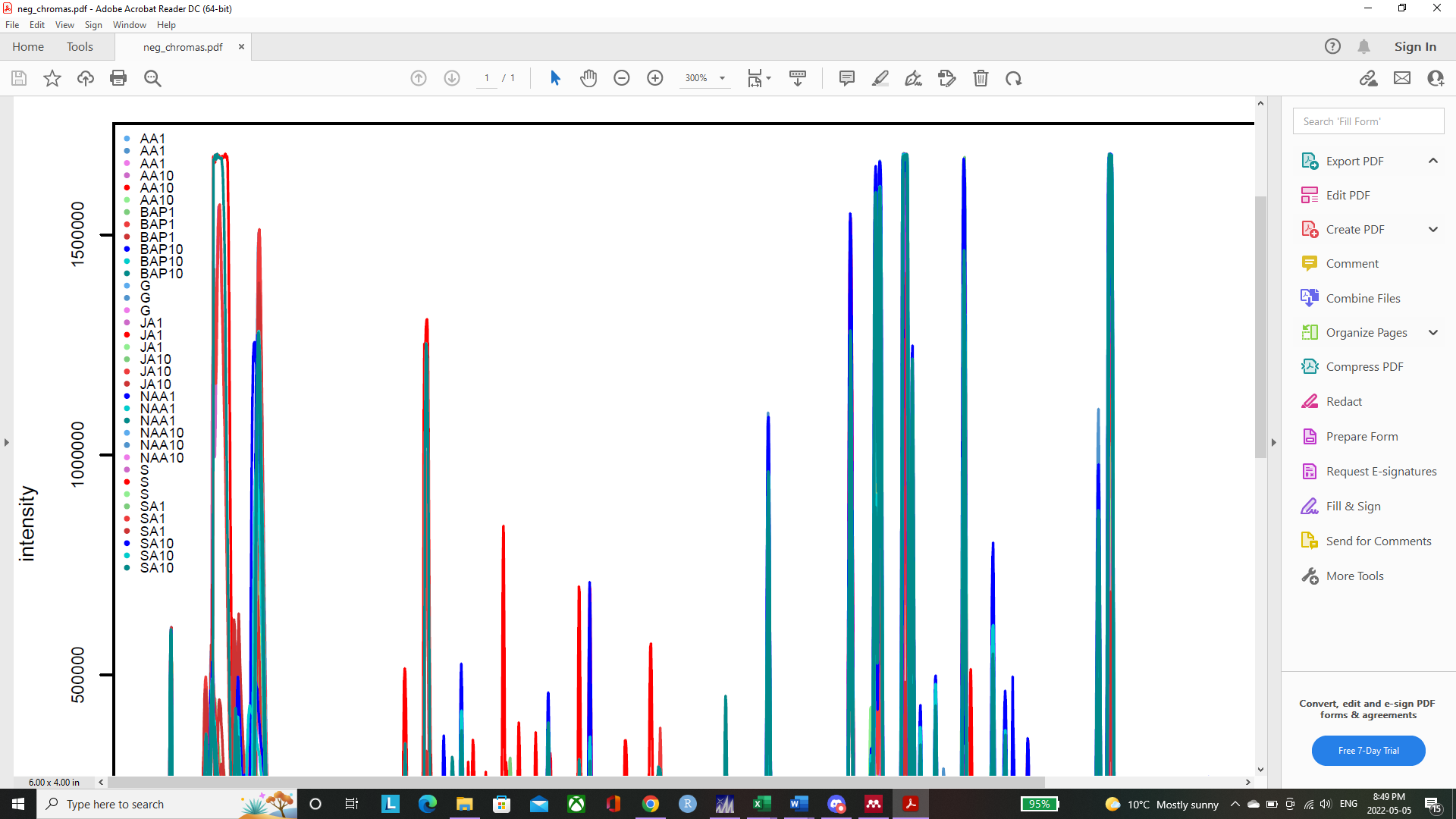
**
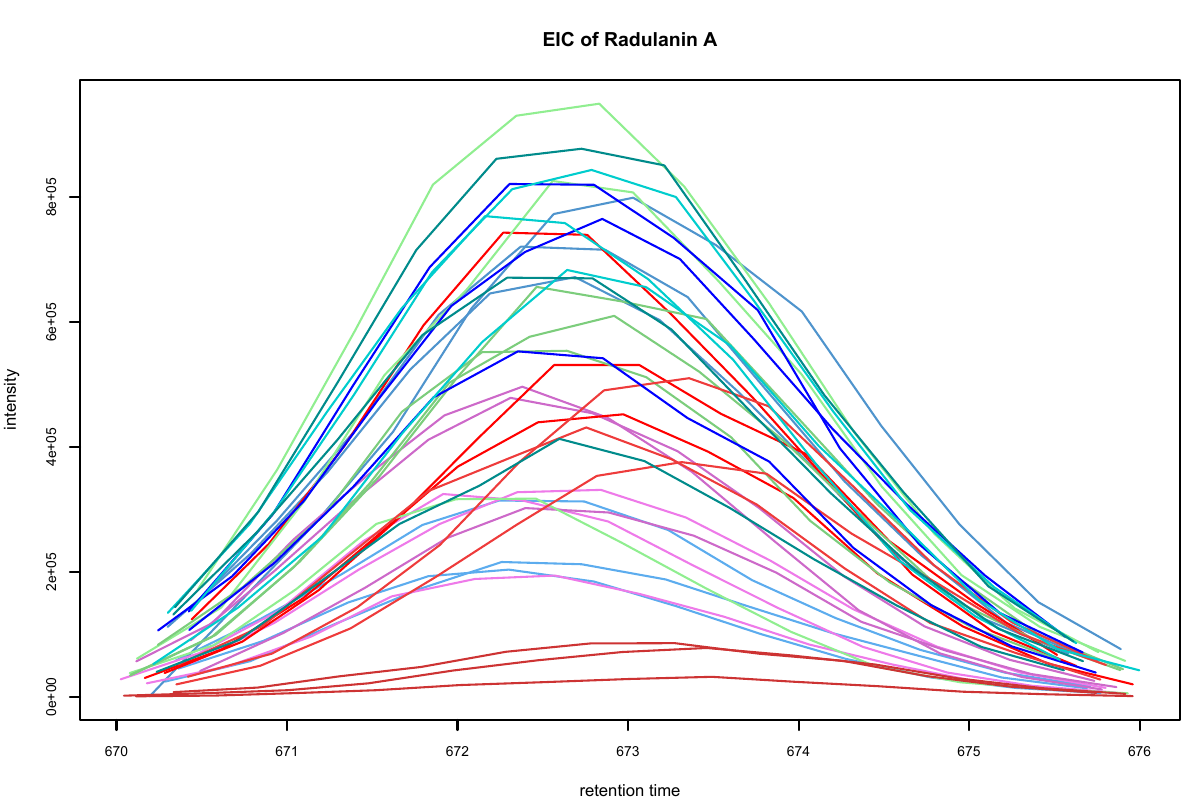
Figure S13.** EIC of Radulanin A in positive mode.


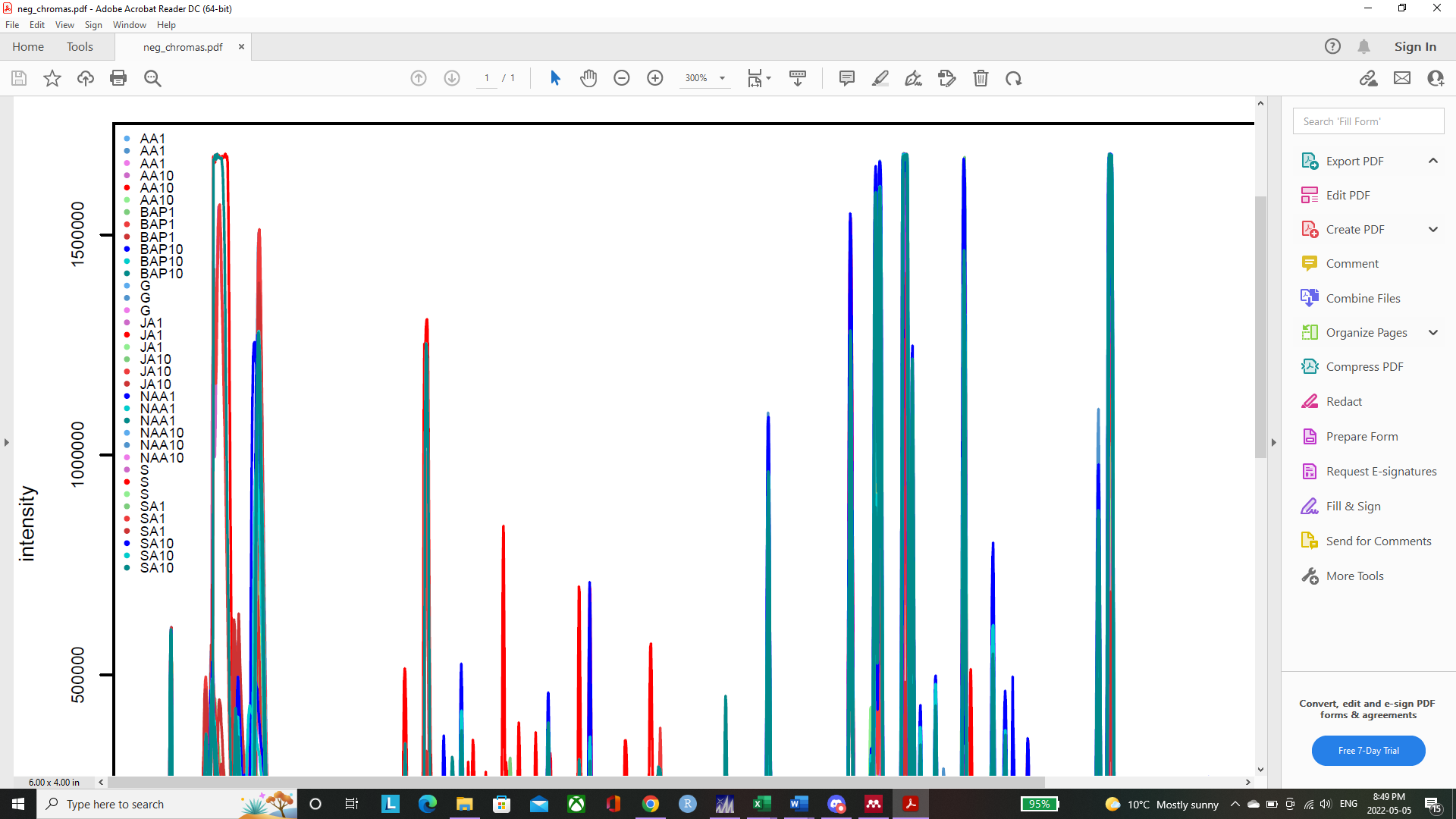
**
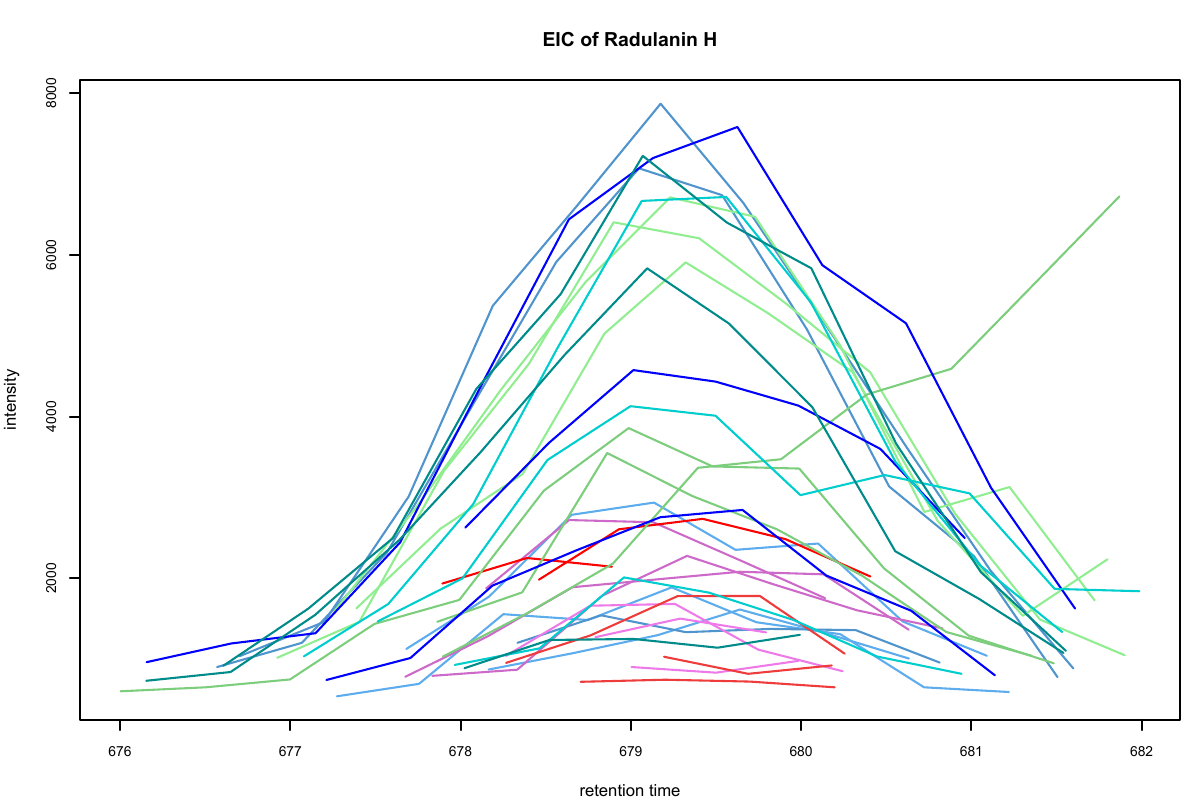
Figure S14.** EIC of Radulanin H in positive mode.


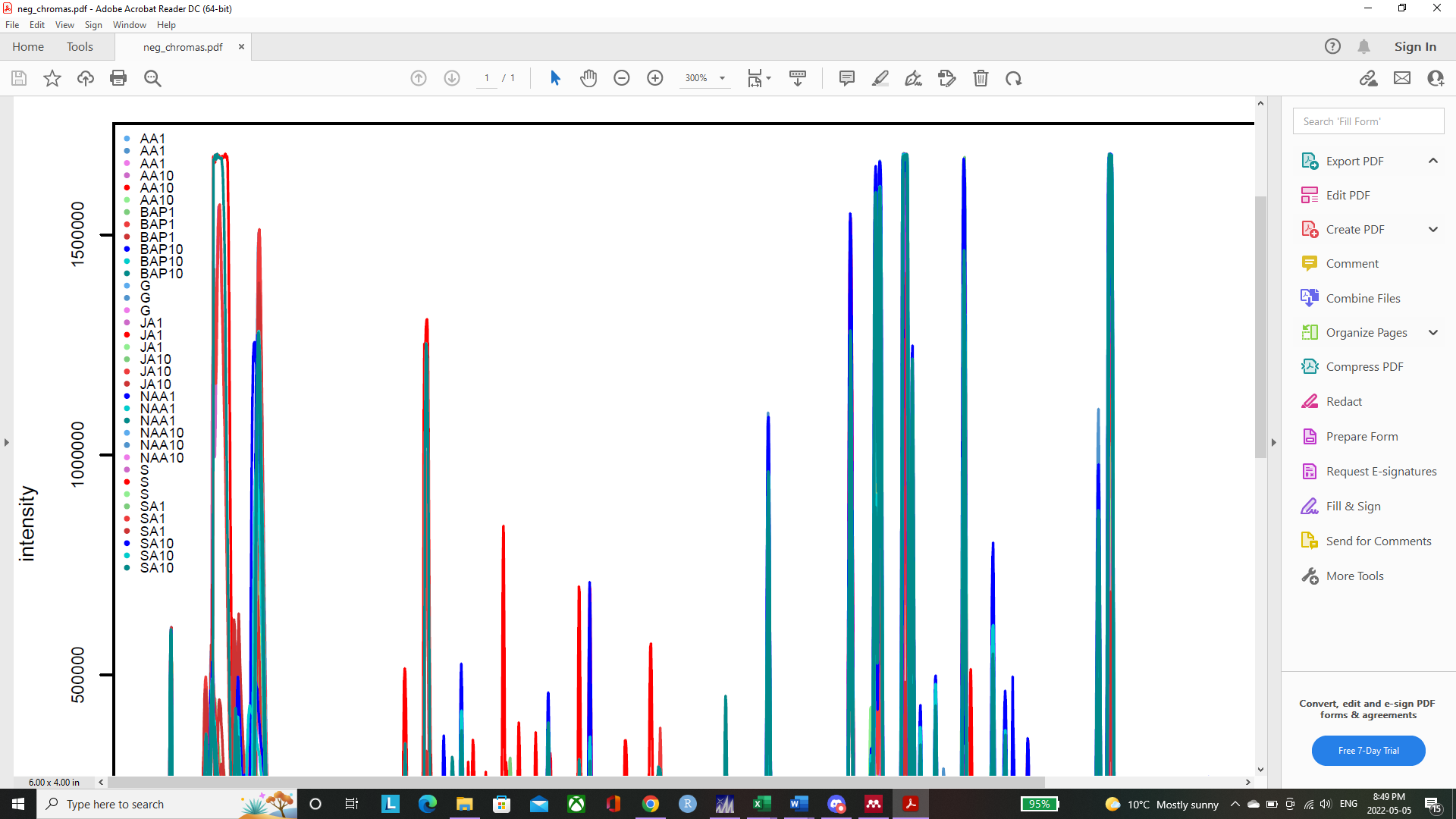
**
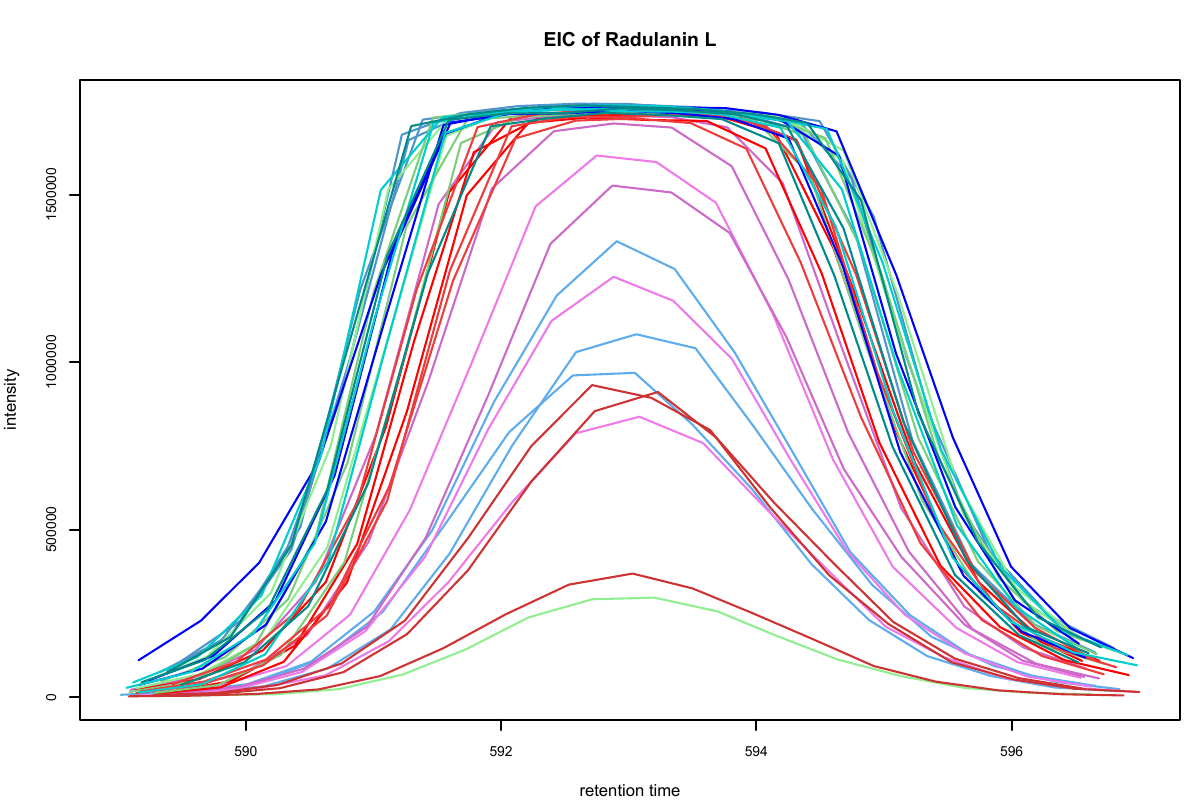
Figure S15.** EIC of Radulanin L in positive mode.


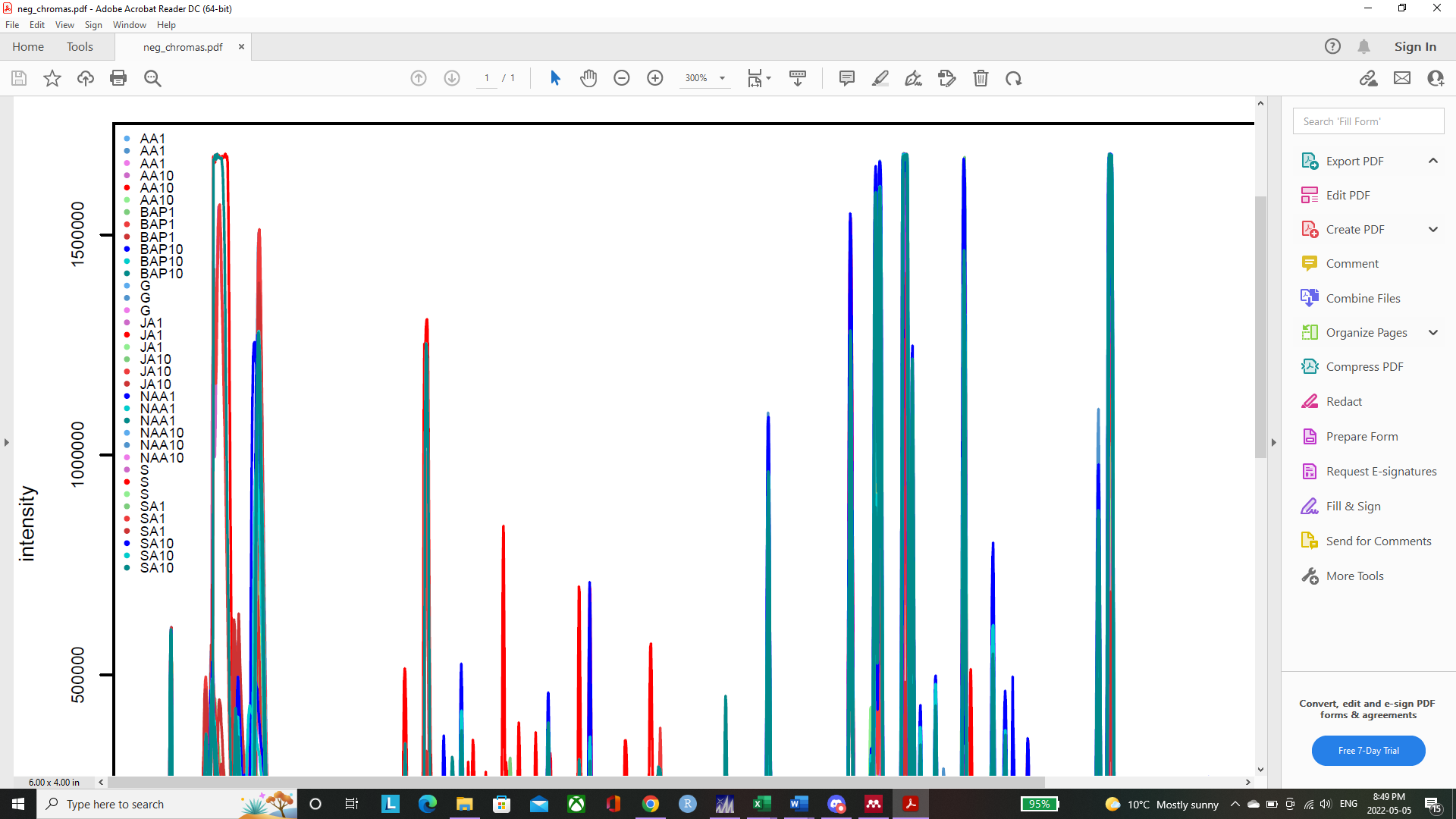
**
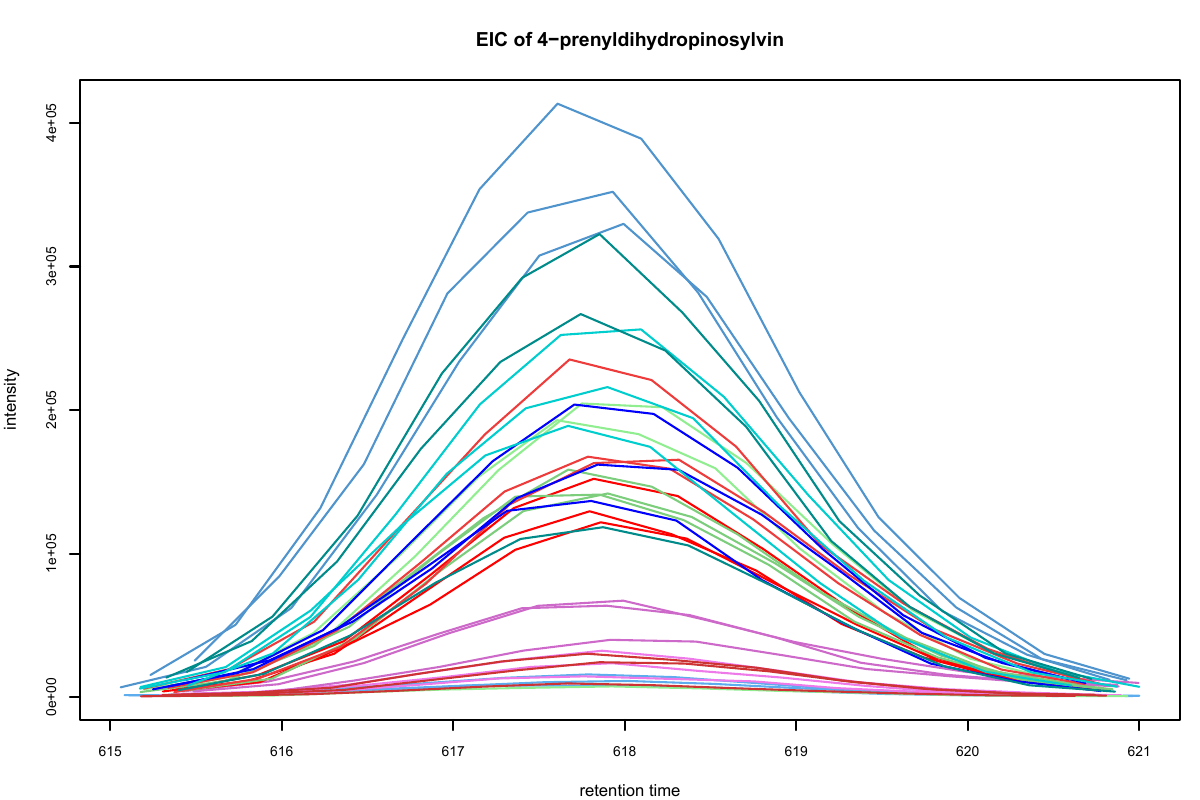
Figure S16.** EIC of 4-prenyldihydropinosylvin in positive mode.


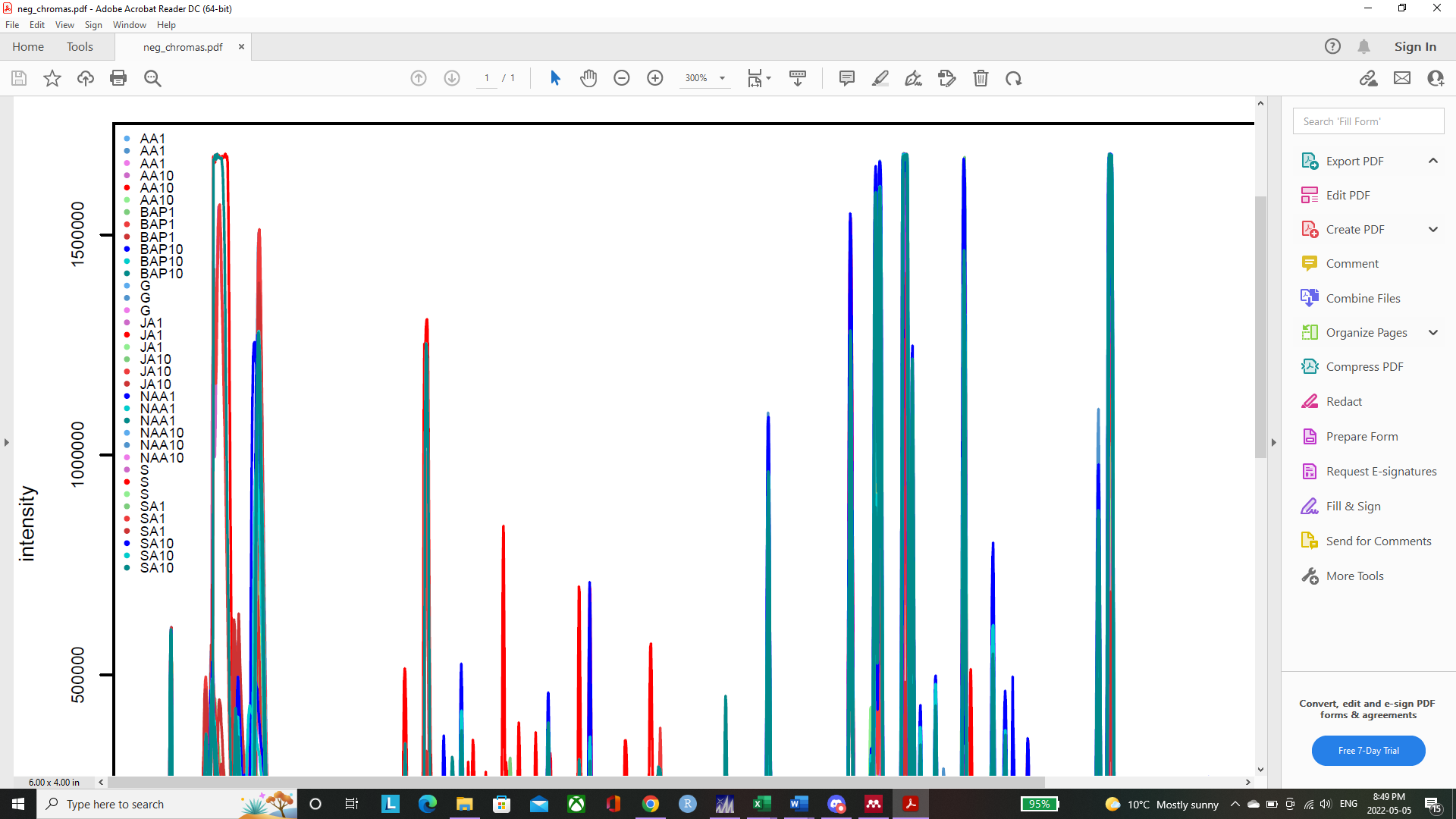
**
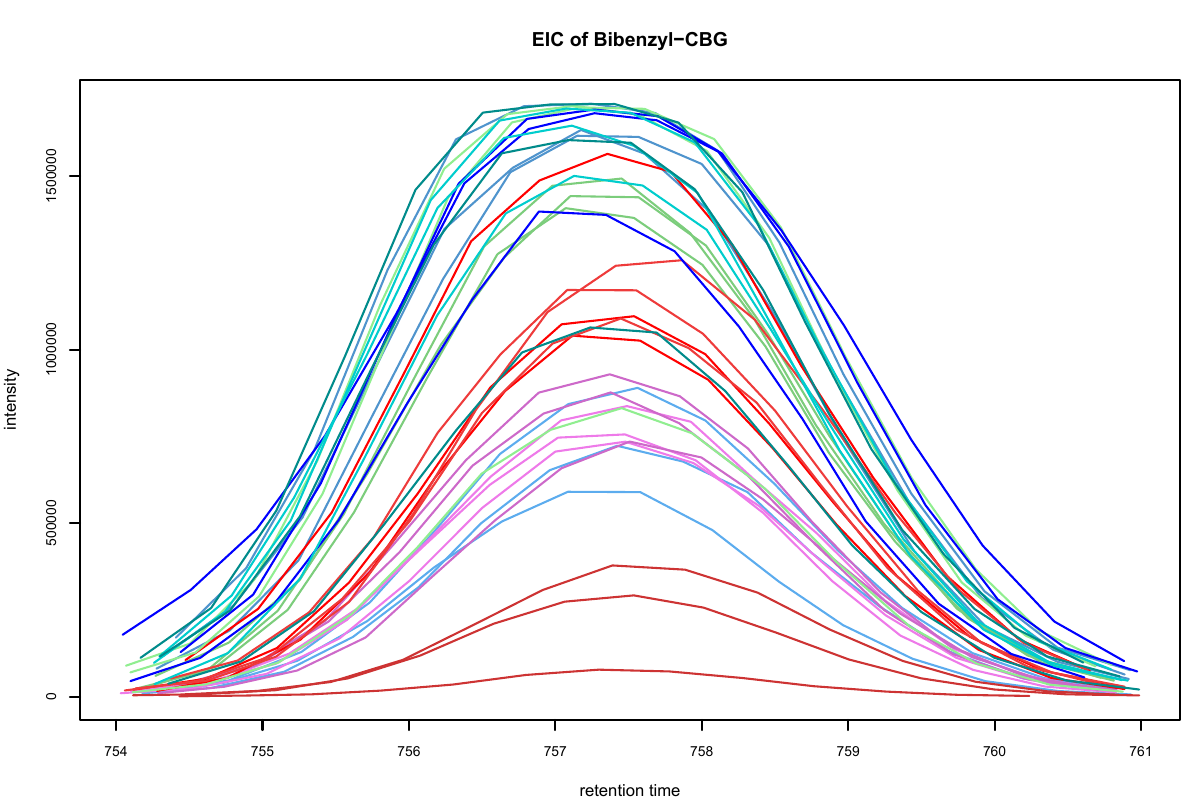
Figure S17.** EIC of 2-(3,7-Dimethylocta-2,6-dienyl)-5-(2-phenylethyl)benzene-1,3-diol in positive mode.


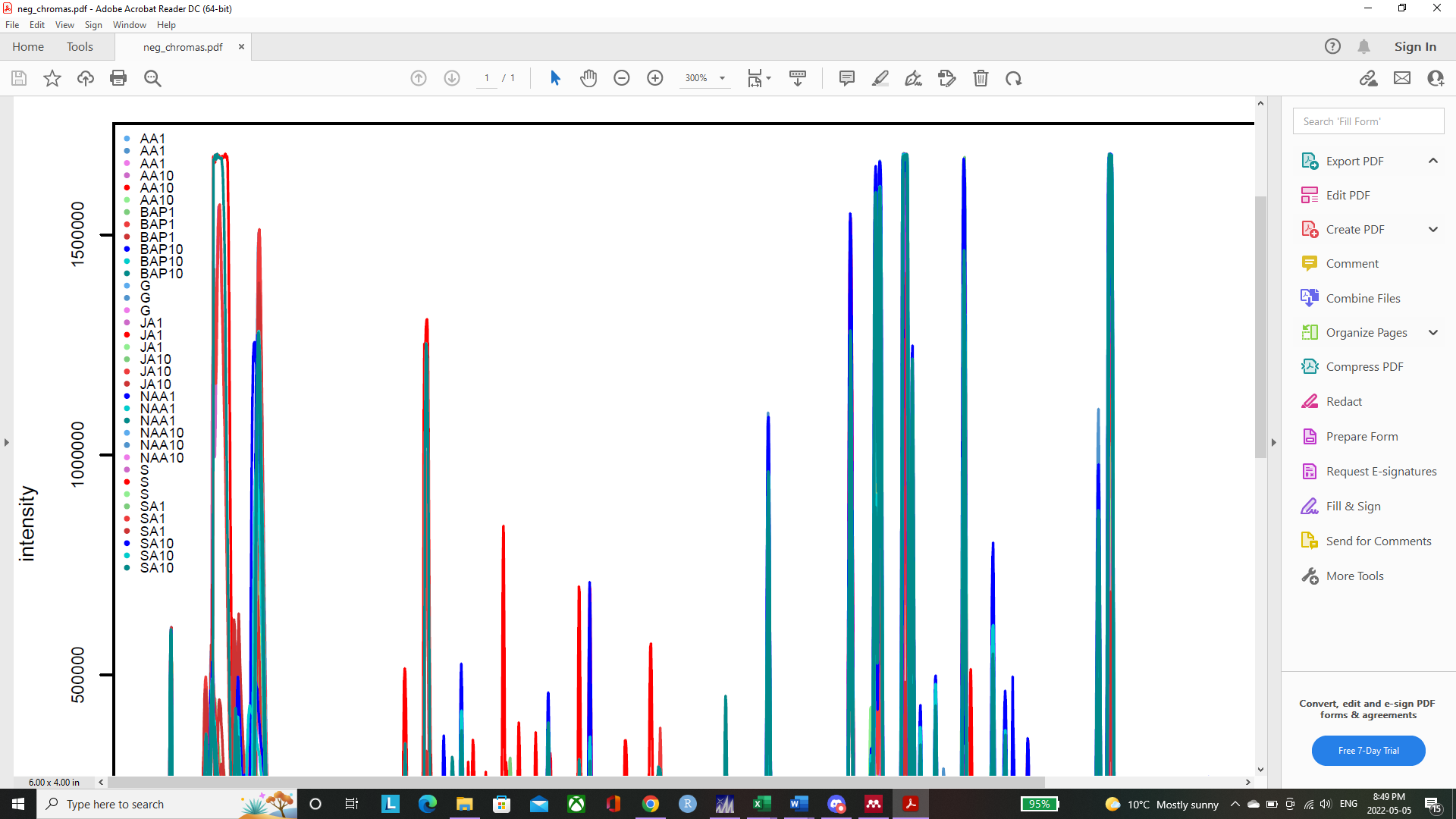
**
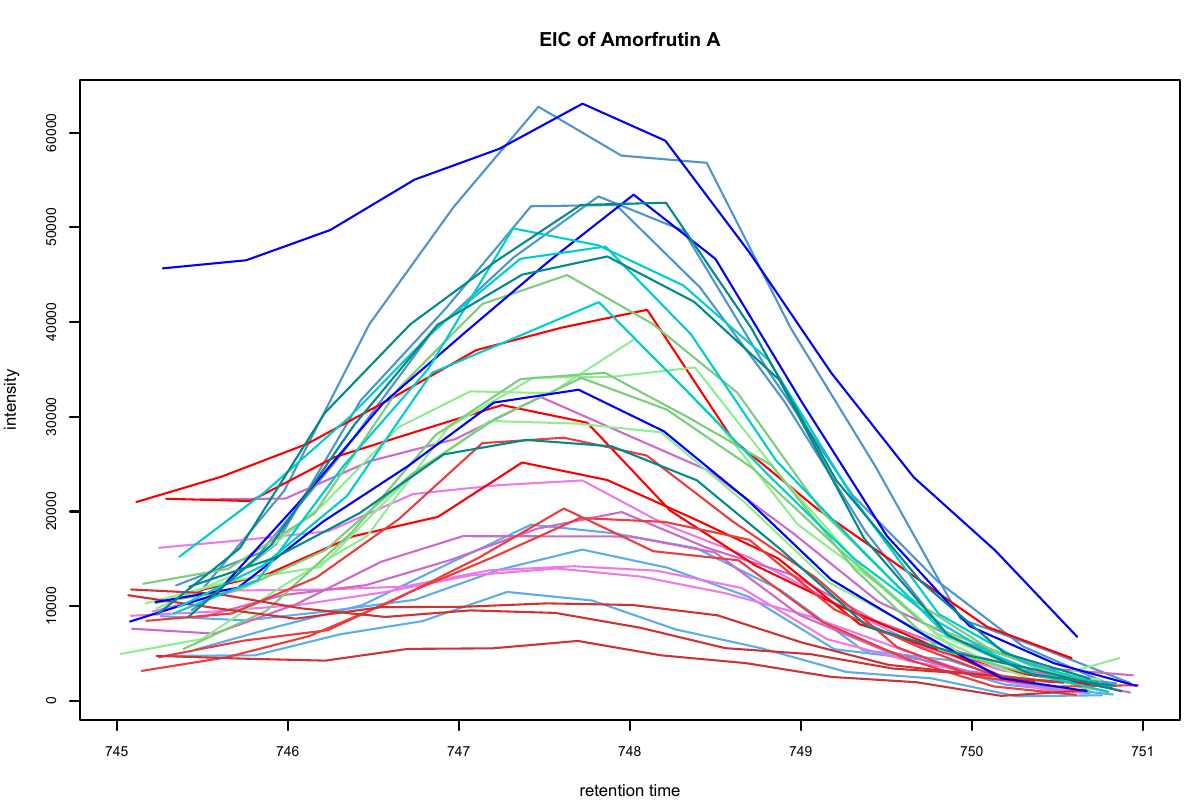
Figure S18**. EIC of 3,5-dihydroxy-6-carbomethoxy-2-(3-methyl-2-butenyl)bibenzyl in positive mode.

- 1. **Data visualization and variance investigation**

**
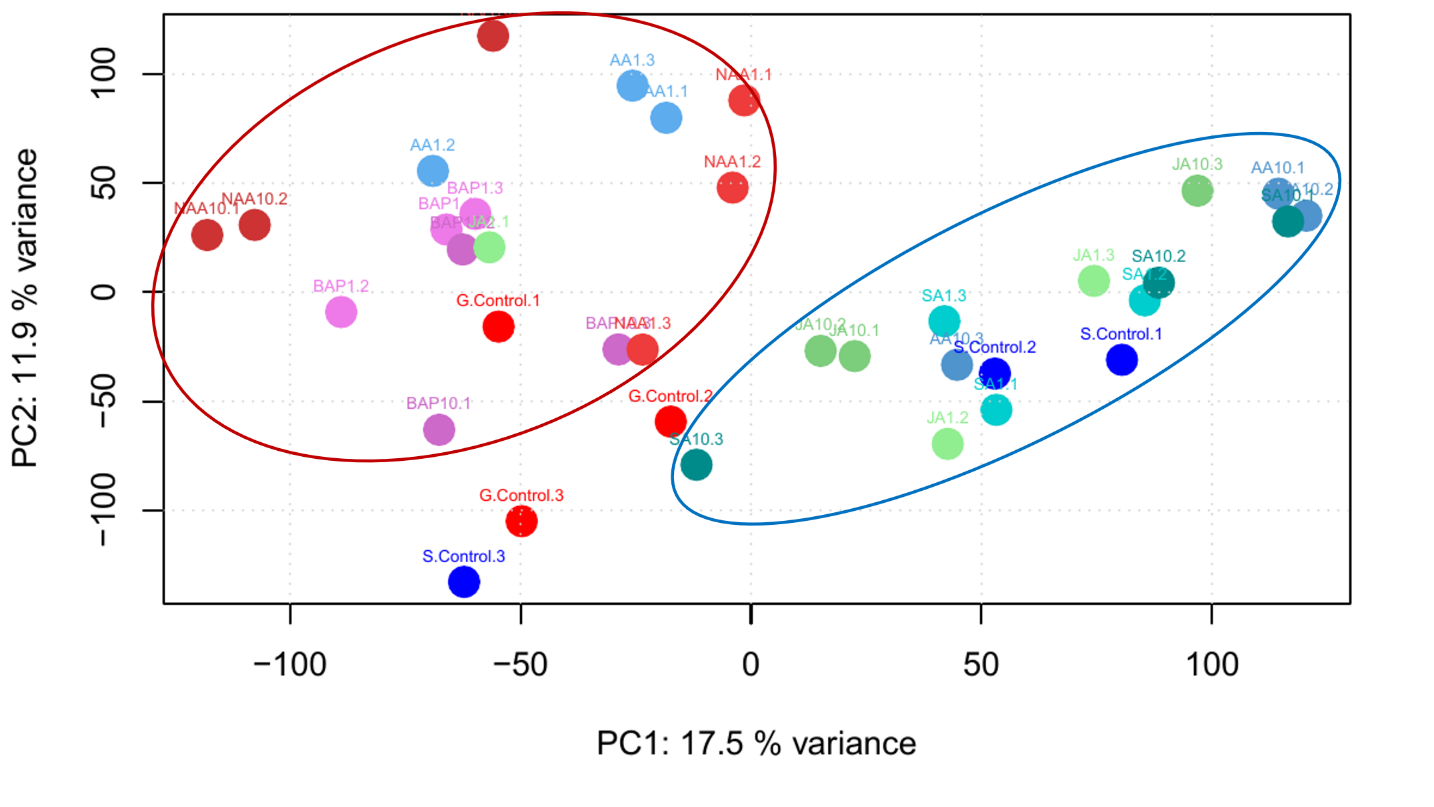
**Data grouping was visualized with PCA plots and potential factors influencing sample variance were explored with variation partitioning.

**Figure S19.** PCA of combined positive and negative MS1 data for all hormone treatments and controls. Circles were manually added with growth treatments (coloured red-pink) circled in red while stress treatments (coloured blue-green) are circled in blue.


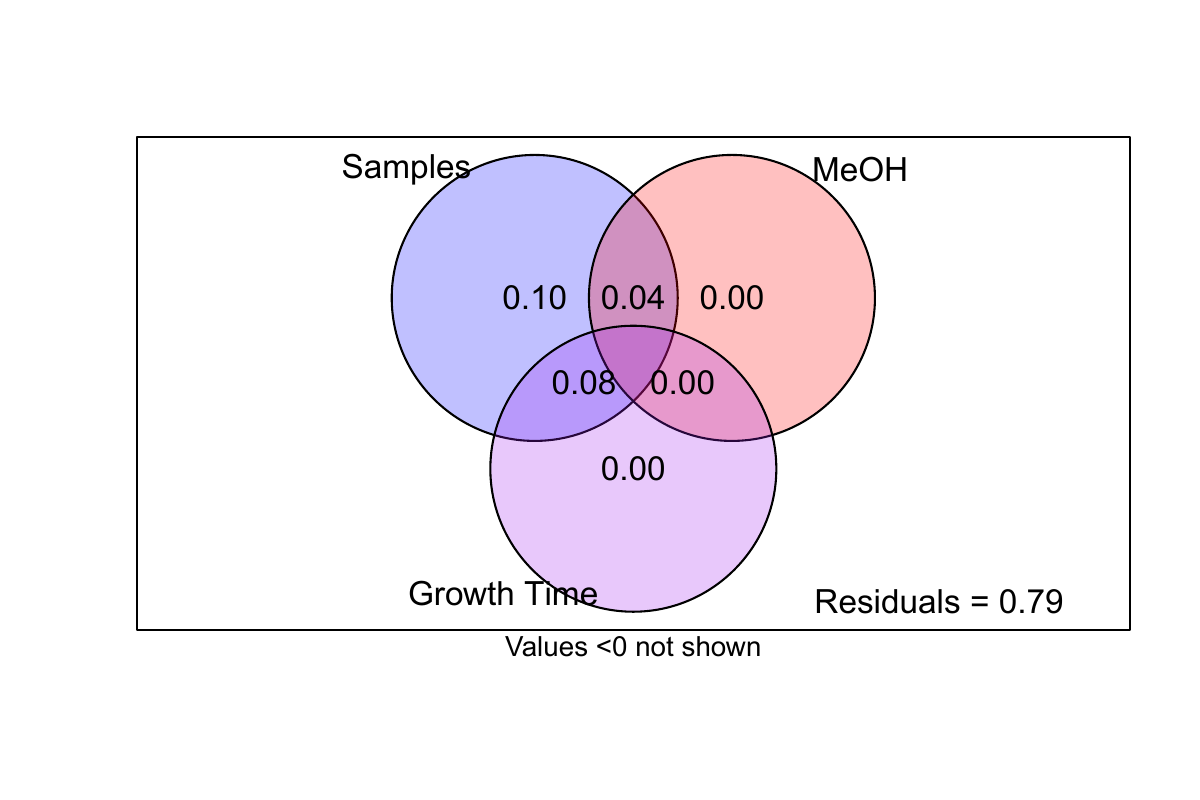
**Figure S20**. Variation partitioning of combined MS1 data for hormone type/growth time (3 vs 4 months) and MeOH concentration per plate. Cumulative explained variance for factor MeOH: 4.0% (4.0% shared with factor samples) and cumulative explained variance for factor Growth Time: 8.0% (8.0% shared with factor samples).

- 1. **Variable selection with BORUTA**


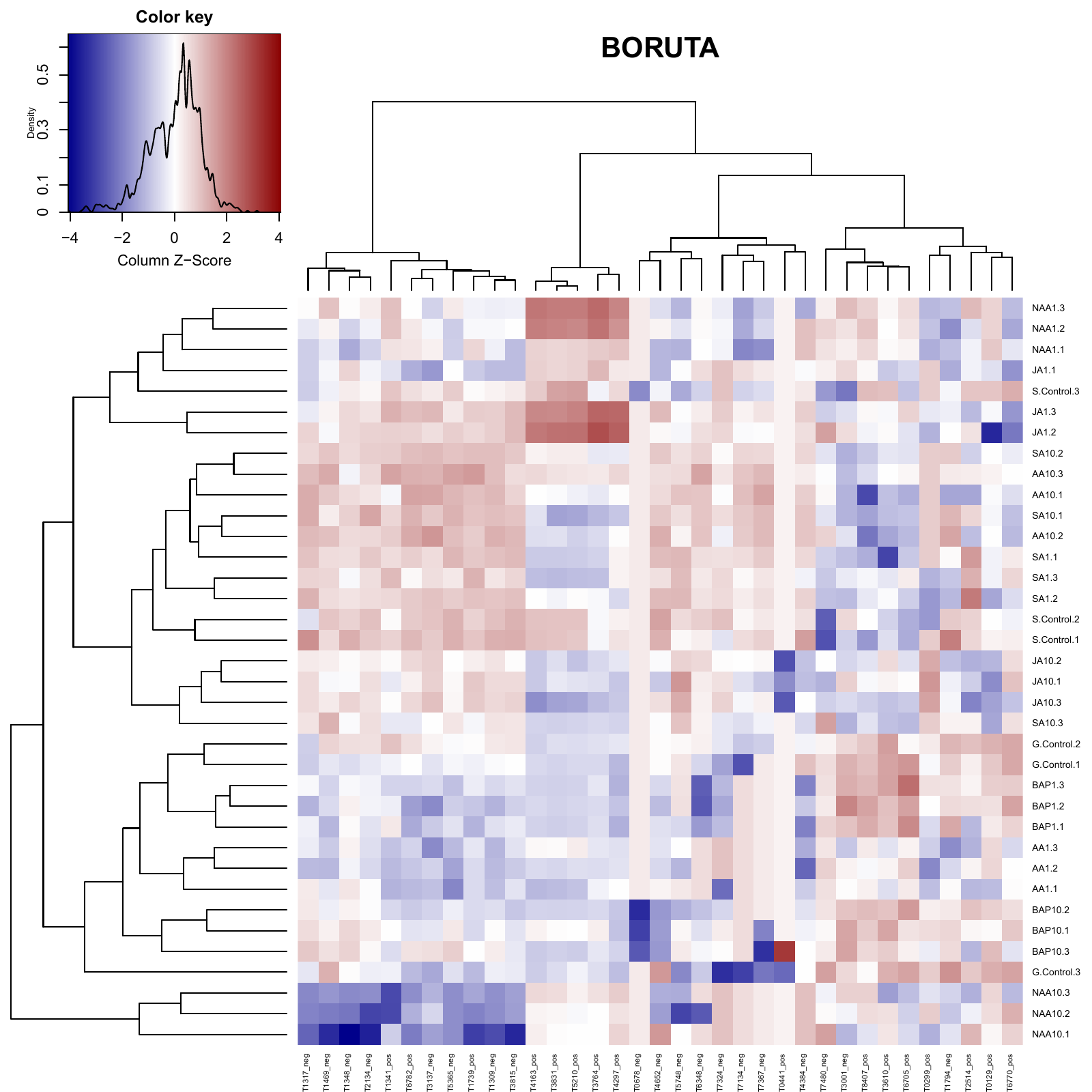
Feature selection using a Random Forest model paired with a BORUTA wrapped algorithm was employed to select features that were responsible for the variation observed in the data.

**Figure S21***.* Heatmap of the selected variables using a BORUTA strategy using the entire combined feature table. The x axis shows the clustering of selected features while the y axis shows the clustering of samples. R^2^ = 1, RMSE = 0, MAE = 0. Selection level: samples (AA1, AA10, BAP1, BAP10, JA1, JA10, NAA1, NAA10, SA1, SA10, SControl, GControl).

**
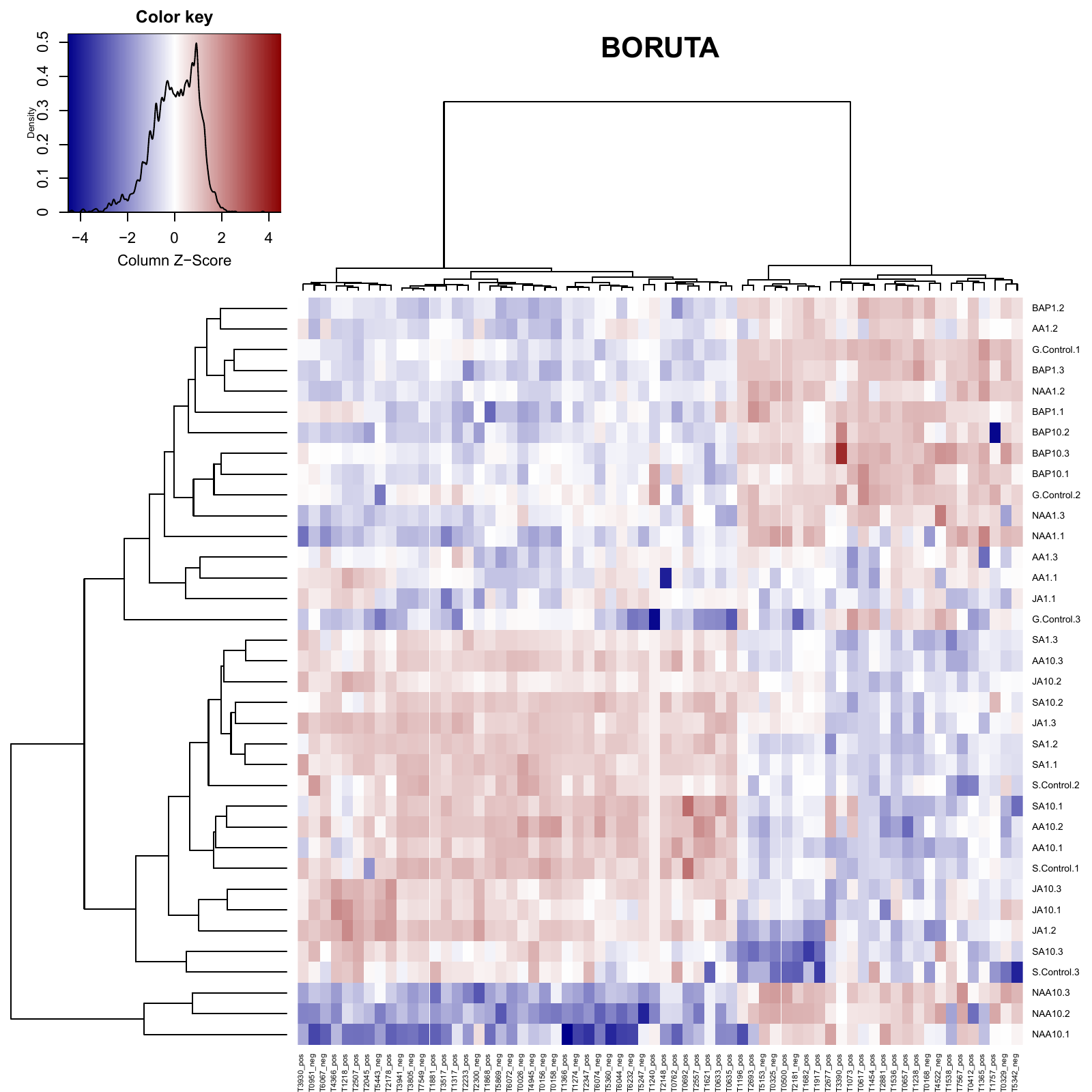
Figure S22.** Heatmap of the selected variables using a BORUTA strategy using only features that had useable MS2 data. The x axis shows the clustering of selected features while the y axis shows the clustering of samples. R^2^ = 1, RMSE = 0, MAE = 0. Selection level: hormone type (stress, growth).

**Detailed Interpretation of Figure 5**

The most detected primary metabolic classes were peptides, amino acids, fatty acids, carboxylic acid derivatives and carbohydrates/carbohydrate conjugates. Six features were classified as peptides and a few significant changes were observed. In the NAA1 and NAA10 treatments a mixed response was observed with an increase in FT4163 and a decrease in FT4254 and FT4497. FT4163 was also found to be downregulated in AA1, JA10, and SA1. The remaining three peptides showed no significant changes with the respect to the control, however FT5410 was only upregulated in BAP while FT4912 was only upregulated in both NAA and BAP. Three fatty acids were identified and two were upregulated in the growth hormone treatments while one (FT1545) was downregulated in AA1. FT1439 showed a significant increase in BAP1, BAP10, NAA1, and NAA10 while FT0761 was upregulated in BAP10, NAA1, and NAA10. Two carboxylic acid derivatives were identified and FT0883 was found to be downregulated in all stress treatments, but none of these changes were significant. Three carbohydrates or carbohydrate conjugates were identified, and no change was observed in FT1105. In FT3098 downregulation in AA1, JA10, SA1, and SA10. FT2415 was identified as a hexose and was downregulated in BAP10 and upregulated in JA10. Five amino acids were identified, however only two features demonstrated significant increases in the NAA treatments. In FT3453, an increase in only NAA1 was observed while in FT0804 an increase in NAA1 and in NAA10 was observed. For the specialized metabolites, the most identified classes were the glycosylated flavonoids, phenols, stilbenes, benzenoids, anisoles, and terpenoids. Three features were identified as glycosylated flavonoids, but no significant changes were observed. Two flavonoids showed minimal changes between treatments while FT4783 was found to be upregulated in all stress treatments and downregulated in all growth treatments. Three phenols and one phenolic glycoside were identified, but the only significant change was the downregulation of FT1717 (a phenol) in NAA10. Two stilbenes were classified, and FT1001 was tentatively identified as 4-(3-Methyl-2-butenyl)-5-phenethylbenzene-1,3-diol. FT1368 was upregulated in both NAA and BAP, however no significant changes were observed in either stilbene. Two terpenoids, classified as a triterpenoid (FT5587) and a terpene lactone (FT1114), were identified and some significant changes were observed. FT1114 was upregulated in NAA10 and FT5587 was downregulated in AA1, JA1, and BAP1. Four anisoles were identified, but only two showed significant changes. FT1431 was reduced in JA10 while FT2606 was reduced in AA1 and AA10. Three benzenoids were classified but only FT6076 showed a significant decrease in BAP10. FT5450 was identified as a lignan or neolignan and was downregulated in SA10.

At the superclass level, a generic chemical classification defined in the ClassyFire chemical ontology (Djoumbou Feunang et al., 2016), the largest groups were the glycosylated compounds, organonitrogen compounds, and the amines. For the glycosylated compounds, three were identified as O-glycosylated compounds and were mostly downregulated while one (FT2016) was identified as just a glycosylated compound and was upregulated in AA1, JA1, and JA10. The three O-glycosylated compounds each showed a distinct trend; FT6067 showed no significant changes, FT4352 was downregulated in AA1, JA10, SA1, and SA10, and FT4290 was upregulated in NAA1, and NAA10. Four organonitrogen compounds were identified, but only two exhibited significant changes with respect to the controls. FT0984 was downregulated in NAA10 while FT1279 was upregulated NAA1 and NAA10 and downregulated in SA1. No significant changes were observed in the two identified amines, but an aralkylamine (FT2110) was significantly downregulated in all stress treatments.

Five of the selected features were tentatively assigned structures based on matching the MS2 fragmentation pattern with those found in the databases called by SIRIUS. One of the identified compounds was IAA-L-Ala, one of the ISTDs added to the samples during the extraction procedure. No significant changes for this feature were observed with respect to the controls. Two features (FT0880 and FT0886) were tentatively identified as BAP and showed significant upregulation in the BAP treated samples. FT0886 was only upregulated in the BAP treated samples while FT0880 showed an increase in only BAP10. An additional increase was also observed in JA1 while a decrease was observed in AA1 and NAA10. GDPhexose (FT5342) was increased in all stress treatments while 4-(3-Methyl-2-butenyl)-5-phenethylbenzene-1,3-diol (FT1001) was upregulated in all growth treatments, but not significantly.

| **Kingdom** | **Superclass** | **Class** | **Subclass** | **Level 5** |
| --- | --- | --- | --- | --- |
| Organic compounds | Phenylpropanoids and polyketides | Stilbenes |  |  |
| Organic compounds | Benzenoids | Benzene and substituted derivatives | 1-hydroxy-2-unsubstituted benzenoids |  |
| Organic compounds | Benzenoids | Benzene and substituted derivatives | Phenol ethers |  |
| Organic compounds | Benzenoids | Phenols | Benzenediols | Resorcinols |

**Table S2.** ClassyFire chemical ontology classification options for bibenzyls
